# Lipidomics Analysis Indicates Disturbed Hepatocellular Lipid Metabolism in *Reynoutria multiflora*-Induced Idiosyncratic Liver Injury

**DOI:** 10.3389/fphar.2020.569144

**Published:** 2020-12-21

**Authors:** Xiaofang Wu, Yating Zhang, Jiaqi Qiu, Ya Xu, Jing Zhang, Juan Huang, Junqi Bai, Zhihai Huang, Xiaohui Qiu, Wen Xu

**Affiliations:** ^1^ The Second Clinical College, Guangzhou University of Chinese Medicine, Guangzhou, China; ^2^ Key Laboratory of Quality Evaluation of Chinese Medicine of the Guangdong Provincial Medical Products Administration, Guangzhou, China; ^3^ Guangdong Provincial Hospital of Chinese Medicine, Guangzhou, China; ^4^ Guangdong Provincial Key Laboratory of Clinical Research on Traditional Chinese Medicine Syndrome, Guangzhou, China

**Keywords:** Polygonum multiflorum, phospholipid metabolism, hepatotoxicity, phosphatidylcholine, phosphatidylethanolamine, Reynoutria multiflora

## Abstract

The root of *Reynoutria multiflora* (Thunb.) Moldenke (syn.: *Polygonum multiflorum* Thunb., HSW) is a distinguished herb that has been popularly used in traditional Chinese medicine (TCM). Evidence of its potential side effect on liver injury has accumulated and received much attention. The objective of this study was to profile the metabolic characteristics of lipids in injured liver of rats induced by HSW and to find out potential lipid biomarkers of toxic consequence. A lipopolysaccharide (LPS)-induced rat model of idiosyncratic drug-induced liver injury (IDILI) was constructed and evident liver injury caused by HSW was confirmed based on the combination of biochemical, morphological, and functional tests. A lipidomics method was developed for the first time to investigate the alteration of lipid metabolism in HSW-induced IDILI rat liver by using ultra-high-performance liquid chromatography/Q-exactive Orbitrap mass spectrometry coupled with multivariate analysis. A total of 202 characterized lipids, including phosphatidylcholine (PC), lysophosphatidylcholine (LPC), phosphatidylethanolamine (PE), lysophosphatidylethanolamine (LPE), sphingomyelin (SM), phosphatidylinositol (PI), lysophosphatidylinositol (LPI), phosphatidylserine (PS), phosphoglycerols (PG), and ceramide (Cer), were compared among groups of LPS and LPS + HSW. A total of 14 out 26 LPC, 22 out of 47 PC, 19 out of 29 LPE, 16 out of 36 PE, and 10 out of 15 PI species were increased in HSW-treated rat liver, which indicated that HSW may cause liver damage via interfering the phospholipid metabolism. The present work may assist lipid biomarker development of HSW-induced DILI and it also provide new insights into the relationships between phospholipid perturbation and herbal-induced idiosyncratic DILI.

## Introduction

Herbal therapies, originated from traditional Chinese medicine (TCM), Indian Ayurvedic medicine, and other traditional medicines, have received increasing attention for their remarkable therapeutic properties; however, there is simultaneously growing concern about the increase in their potential side effect. Herbal-induced liver injury (HILI), presenting an increasing trend, has recently become a challenging issue (Li et al., 2007; Björnsson et al., 2013; Teschke and Eickhoff, 2015).

According to experiences of traditional Chinese medicine, the root of *Reynoutria multiflora* (Thunb.) Moldenke (He Shou Wu, HSW) is one of the beneficial and tonic herbs for treatment of chronic liver and kidney diseases (Li et al., 2016), alopecia, and age-related cognitive dysfunction (Park et al., 2017). A significant number of liver injury cases and even casualties caused by HSW have, however, been reported from more than 30 countries in the recent decade (Jung et al., 2011; Wang et al., 2015). HSW has consequently been regarded as the top herb associated with HILI in China, accounting for approximately 30% of HILI cases (Wang et al., 2018b).

The underlying mechanisms of HSW-induced liver injury remain unclear. A part of clinical cases have reported that it appears to be idiosyncratic, without regard to its dosage and herbal processing (Park et al., 2001; Jung et al., 2011; Dong et al., 2014). Idiosyncratic drug-induced liver injury (IDILI) is a rare reaction among individuals exposed to those drugs inducing liver injury. Although the pathogenesis of IDILI is poorly understood, it has been considered to be associated with genetics, host susceptibility, and environmental factors. Non-genetic factors includes age, sex, chronic liver diseases, human dysimmunity, and drug–drug interaction resulting from polypharmacy (Uetrecht, 2019). Previous works have identified a close affinity between immune stress and drug idiosyncrasy (Deng et al., 2009; Beggs et al., 2014). A mild immune-stimulated idiosyncratic DILI rodent model induced by bacterial lipopolysaccharide (LPS) has been created and applied to evaluation idiosyncratic DILI properties of some drugs and herbs (Liguori et al., 2010). The idiosyncratic characteristic of HSW-induced liver injury has been confirmed from a mild immune-stimulated idiosyncratic DILI rodent model induced by LPS (Tu et al., 2019); *cis*-stilbene glucoside, one of the major compounds of HSW, was found to induce immunological idiosyncratic hepatotoxicity through suppressing PPAR-γ in this rodent IDILI model (Li et al., 2017a; Meng et al., 2017). Untargeted metabolomics studies (Li et al., 2016; Tu et al., 2019) have indicated that HSW-induced liver injury altered glycerophospholipid metabolism, the tricarboxylic acid cycle, and sphingolipid metabolism in the LPS induced IDILI rat model. These studies implied that lipid metabolism disorder might be involved in HSW-induced liver injury.

Lipids are a general group of essential components in living cells, among which phospholipids, the main components of biomembranes, play pivotal functions in membrane-mediated cell signaling, maintaining cell membrane homeostasis, cellular migration and proliferation, apoptosis, and inflammation. In hepatocytes, phosphatidylcholine (PC) and phosphatidylethanolamine (PE) are the two most abundant phospholipids (Ming et al., 2017). Previous lipidomics studies have shown that disturbances of lipid metabolism, including increase in the contents of PC and PE species (Ming et al., 2017) as well as marked reduction of sphingomyelin (SM) (Xu et al., 2019), were associated with liver injury induced by acetaminophen and valproic acid, respectively. Besides, ceramide (Cer) metabolism was significantly altered by three idiosyncratic drugs (Nimesulide, Nefazodone, and Trovafloxacin), which may induce endoplasmic reticulum (ER) stress and activate the JNK pathway in a HepG2 cell model (Jiang et al., 2017). So far, there have been very few lipidomics studies focusing on the lipid metabolism abnormality associating with herbal-induced liver injury (HILI) (Su et al., 2020). Clinical cases and copious *in vivo* toxicological trials revealed that liver biopsies of HSW-exposure patients or rats had marked with mixed inflammatory cell infiltration and steatosis (Li et al., 2007). Lipid alteration of the hepatocytes induced by HSW has been frequently observed in toxicological or pharmacological studies (Wang et al., 2012; Pei et al., 2014; Jiang et al., 2015). Our preliminary untargeted metabolomics research also demonstrated that two main metabolic pathways were involved, namely, phospholipid metabolism and arachidonic acid metabolism pathways, in a rat model induced by high dosage HSW for one month (unpublished results). Nevertheless, the targeted impacts of HSW exposure on hepatic lipid metabolism have not yet been explored.

Lipidomics is an effective tool to inspect variation in endogenous lipids metabolism by integrating an advanced analytical and multivariate statistical strategy. Liquid chromatography coupled with mass spectrometry (LC-MS)-based lipidomics usually consists of untargeted and targeted approaches, each having their own advantages and disadvantages (Xuan et al., 2018). The untargeted lipidomics, which used to be performed by using high resolution MS, enable us to globally cover many lipid classes in biological samples. The targeted lipidomics strategy, which is conventionally executed on a triple quadrupole (QQQ) mass spectrometer in multiple reaction monitoring (MRM) mode, provide a result with good repeatability, sensitivity, and a wide linear dynamic range (Zhang et al., 2020). Pseudotargeted lipidomics, firstly proposed by Xu et al. (Chen et al., 2013), combines the advantages of both targeted and untargeted strategies (Cao et al., 2020). Both known and unknown metabolites in samples can be measured by using the retention time locking-selected ions monitoring, which offers an efficient means to semi-quantitatively investigate endogenous lipids in different matrices and has been applied to discovery of diseases biomarkers (Wang et al., 2018a; Li et al., 2020).

In the present study, our aim was to globally profile the variations in the level and/or in the composition of lipid species and to explore the specific lipid biomarkers in HSW-induced IDILI rats. An untargeted and pseudotargeted combined lipidomics strategy based on ultra-high-performance liquid chromatography coupled with Q-exactive hybrid Orbitrap mass spectrometry (UHPLC-QE-Orbitrap-MS) was performed to analysis the endogenous lipids metabolites in the LPS-induced IDILI rat model. To the best of our knowledge, it is the first lipidomics study to explore the underlying mechanisms of HSW-induced liver injury, which is essential for a better understanding of the relationships between lipid perturbation and herbal-induced IDILI.

## Materials and Methods

### Chemicals and Materials

LC-MS grade acetonitrile, methanol, and 2-propanol were purchased from Merck (Darmstadt, Germany). Formic acid (LC-MS grade) was obtained from Thermo Fisher Chemicals (Pittsburg, PA, United States). Lipopolysaccharide (LPS) and 2,6-di-tert-butyl-4-methylphenol (BHT) was purchased from Sigma-Aldrich (St. Louis, MO, United States). LC grade dichloromethane was obtained from Guangzhou Chemical Reagent (Guangzhou, Guangdong, China). Assays kits for detection of serum alanine aminotransferase (ALT), aspartate aminotransferase (AST) and total bile acid (TBA) were purchased from Jiancheng Biological Technology, Co., Ltd. (Nanjing, Jiangsu, China). INOS, IL-6, COX-2, and HMGB-1 ELISA arrays kits were provided by CUSABIO Co., Ltd. (Wuhan, Hubei, China). Internal standard compound lysoPE (14:0) was purchased from Avanti Polar Lipids, Inc. (Alabaster, Al, United States).

The root of *Reynoutria multiflora* (HSW, 190,501), was obtained from Kangmei Pharmaceutical Co., Ltd. (Puning, Guangdong, China). The dried sample was extracted twice by hot reflux of eight-times volumes of 70% ethanol-water for 1 h. The combined extract was concentrated under negative pressure at 50°C and then subjected to freeze drying to yield the HSW extract. The main constituents of the sample were analyzed by using a LC-MS approach, which was expatiated in the supplementary file.

### Animals

Male specific-pathogen-free (SPF) grade Sprague-Dawley (SD) rats were purchased from Animal Center of the Southern Medical University (Certification number: 44002100020055) with weights of 180 ± 5 g. All procedures on animals complied with the guideline and their care is under supervision and inspection of the laboratory animal ethics committee of Guangdong Province Hospital (Guangdong, China). Prior to the experiments, all animals were accommodated to the experimental environment for 3 days, where 12 h of circadian circulation were provided and rats had free access to a standard diet and water.

### Treatment of Rats

A mild immune-stimulated idiosyncratic DILI model was constructed via pre-stimulation of rats with lipopolysaccharide (LPS) (Uetrecht, 2019). Animals were randomly divided into six groups with 25 rats in each groups: the normal control group (A, control); the LPS-induced model group (B, LPS); the rats treated with HSW at dose of 2 g/kg/day (equivalent of raw herb) group (C, L-HSW); the rats treated HSW with higher dose of 10 g/kg/day (equivalent of raw herb) (D, H-HSW); the LPS model rats treated with dose of 2 g/kg/day HSW (equivalent of raw herb) (E, LPS + L-HSW); and the LPS model rats treated with higher dose of 10 g/kg/day HSW (equivalent of raw herb) (F, LPS + H-HSW). LPS (2 mg/kg) or saline was injected into the tail vein of rats using standard techniques, and 2 ml of blood was collected from the orbit after 2 h, 24 h and 5 days, respectively. The animals were intragastrically administered different doses of HSW or saline for 7 days without interruption. Food and water were available to all rats ad libitum throughout the experiment. On the eighth day, the rats were anesthetized with 10% chloral hydrate (0.3 ml/100 g), and blood was collected from the inferior vena cava by heparin sodium blood collection tubes. The livers were isolated from the rats immediately after sacrifice for histopathological evaluation. The serum samples separated from the gathered blood were utilized for biochemical tests.

### Biochemical Analysis

Liver function was assessed by determined the activities of ALT, AST, and TBA, which were measured with corresponding kits. The levels of four serum inflammatory cytokines iNOS, IL-6, COX-2, and HMGB-1 were evaluated by using ELISA assay kits according to the manufacturer’s instructions.

### Histopathological Analysis of Liver Tissue

Liver Tissues from the same site of rats were fixed with 10% neutral formalin for more than 24 h and then embedded in paraffin. The embedded sections were cut into 4 µm thickness and stained with hematoxylin and eosin (H&E) for microscopic examination. Qualitative evaluation of histological features, including general hepatocellular morphological characteristics, steatosis, inflammatory infiltration, hepatocellular necrosis, was conducted referring to the DILI Pathological Scoring System (DILI-PSS) (Hu, 2012) and nonalcoholic liver disease (NAFLD) Scoring System (the Pathology Committee of NASH Clinical Research Network, NASH-CRN) (Zhou et al., 2007).

### Liver Tissue Preparation for Lipidomics Analysis

The extraction of lipid metabolites was based on the Folch method with a slight modification in which dichloromethane: methanol (2:1, v/v) was used as the base extraction solution instead of chloroform: methanol. Each homogenization tube, containing 50 mg of liver tissue, 20 ng internal standard LysoPE (14:0), and several small ceramic beads, were homogenized in a homogenizer by adding 1 ml of dichloromethane: methanol (2:1, v/v) mixed solvent containing 10 μM BHT. The homogenates were centrifuged at 13,000 rpm for 15 min at 4°C. The supernatants were dried with nitrogen and stored at −80°C until analysis. In the redissolution process, the dried samples were dissolved in 200 μL of methanol: isopropanol (1:1,v/v) solution, subjected to vortexing for 30 s, and centrifuged at 15,000 rpm at 4°C for 15 min to collect the supernatants. All the sample preparation procedures were carried out in ice-bath.

### Instrumentation and Conditions

The chromatographic separation of each lipid sample was performed in an U3000 UHPLC (Thermo fisher, USA) with a Waters HSS T3 UPLC™ (2.1 × 100 mm, 1.7 μm) column. The separation parameters were optimized with regards to the composition of the mobile phase and elution program as follows. The linear gradient was adopted in elution with the mobile phases of solvent A: methanol: acetonitrile: water (1:1:1, v/v) containing 5 mM ammoniumformate and 0.1% formic acid, and solvent B: isopropanol: acetonitrile (9:1, v/v) containing 5 mM ammonium formate and 0.1% formic acid. The optimal gradient elution program was as follows: 0% B for 5 min, then linearly increased to 40% B at 5 min, to 60% B at 9 min, to 95% B at 15 min and maintained for 10 min, followed by 5 min equilibration. The elution flow rate was set at 0.20 ml/min, the column was held at 30 °C, and the temperature of the sample tray was set at 4 °C.

Eluted lipids were analyzed by a Q-Exactive (QE) hybrid Orbitrap mass spectrometry (Thermo Fisher Scientific, USA) with an electrospray ionization source (ESI). The MS was manipulated with voltage of 3.7 kV in negative mode, collected in the full scan range of *m/z* 120–1,450. Other main parameters of ESI were set as follows: sheath gas: 35 psi; aux gas: five psi; capillary temperature was 350 °C, and probe heater temperature was 320 °C. External mass calibration was carried out using the MS manufacturer’s guidelines before sample tests. All samples were analyzed in a random order, and a quality control (QC) sample, composed of an aliquot of each sample, was inserted into the batch once every 10 sample tests to evaluate the repeatability and stability of analysis.

### Data Processing and Lipid Identification

For non-targeting lipids, the high-accuracy *m/z* values extracted by Compound Discoverer™ (Thermo Fisher Scientific, United States) were primarily annotated by searching in the LIPID MAPS database (http://www.lipidmaps.org/) and in-house database. MS^2^ Characteristic ions were used to further confirm the identification of lipids based on the distinct fragmentation pathways in Q-exactive (QE) Orbitrap MS (Narváez-Rivas and Zhang, 2016; Narváez-Rivas et al., 2017). The mega MS data were preprocessed for peak detection, alignment, correspondence, and normalization by using XCMS package in R language (v3.6.1) platform (Smith et al., 2006). The data matrix was then imported into SIMCA-P+ (v14.1, Umetrics, Umeå, Sweden) for multivariate statistical analysis. Unsupervized Principal components analysis model (PCA) and supervised orthogonal partial least-squares-discriminate analysis model (OPLS) were applied to identify the group discriminators for three groups of liver-detected features.

In the pseudotargeted lipidomics analysis, the relative intensities of targeted lipidome were unbiased extracted by using the Quan Browser model of xcalibur 3.1 (Thermo fisher, USA) in a high-resolution, accurate-mass selected (HR/AM) way. The generated quantitative data were processed for multivariate statistical analysis in the same way. Heat map were generated in R language (v3.6.1) by using a pheatmap package (https://cran.r-project.org/web/packages/pheatmap/index.html).

### Statistical Analysis

All numerical data are shown as mean ± or +standard deviation. Significant differences between groups (*p* value) were evaluated using Graphpad Prism 8.0 software. The differences in the data were tested for normality and homogeneity of variance firstly and determined using one-way analysis of variance (ANOVA) followed by Dunnett’s *t* test. If violation of normality and homogeneity of variance was observed, Kruskal-Wallis test was used. *p* value less than 0.05 was regarded as significance variation.

## Results and Discussion

### Identification of the Chemical Compositions of HSW

The chemical components of HSW were globally investigated in our previously study (Qiu et al., 2013). Generally, stilbenes and anthraquinones are regarded as the main constituents of HSW. In the present study, the HSW sample was analyzed by an UHPLC coupled with a high-resolution Orbitrap MS. A LC-MS chromatogram of the HSW sample is shown in Supplementary Figure S1. Based on the high resolution precursor ions and their characteristic fragment ions, the 16 main peaks were identified as citric acid, procyanidin B, gambiriin A, mono-*O*-galloylprocyanidin, 2,3,5,4′-tetrahydroxy -silbence-2,3-glucoside, 2,3,5,4′-tetrahydroxysilbence, 2,3,5,4′-tetrahydroxysilbence-2 -(galloyl)-glucoside, 2,3,5,4′-tetrahydroxysilbence-2-(acetyl)-glucoside, 2,3,5,4′ -tetrahydroxysilbence-2-(galloyl)-glucoside, citreorosein-*O*-glucoside, 2,3,5,4′-tetra -hydroxy silbence-2-(coumaro-yl)-glucoside, 2,3,5,4′-tetrahydroxysilbence- (feruloyl) -glucoside, torachrysone-8-O-glucoside, emodin-8-*O*-glucoside, emodin-8-*O*-(6′-O -malonyl)-glucoside, and emodin, respectively. The MS information were listed in Supplementary Table S1.

### Evaluation of the Liver Injury Induced by HSW

Previous studies indicated that mild immune stimulation (MIS) can promote the susceptibility of IDILI (Mohamed, 2013) and the LPS-induced IDILI model have been successfully applied to investigate IDILI caused by HSW (Fan et al., 2015; Tu et al., 2019) and other herbs/drugs (Deng et al., 2009). As depicted by Li (Li et al., 2015), the double clinical equivalent dose of HSW (1.08 g/kg/day) caused significant liver injury in MIS model rats. Herein the dosage of HSW was optimized. Based on our preliminary tests, oral administration of HSW from 2 g/kg/day (4-fold clinical equivalent dose) to 10 g/kg/day did not cause liver damage. We therefore selected two dosages at 2 g/kg/day and 10 g/kg/day, respectively, which are lower than Tu’s study (Tu et al., 2019). The result showed that neither consecutive treatments of HSW for 7 days nor single dose of LPS caused evident liver injury in rats. Liver injury in groups of LPS + L-HSW and LPS + H-HSW were, however, confirmed by combination of biochemical, morphological and functional tests, indicating the LPS-induced IDILI model for HSW was successfully developed.

**FIGURE 1:**
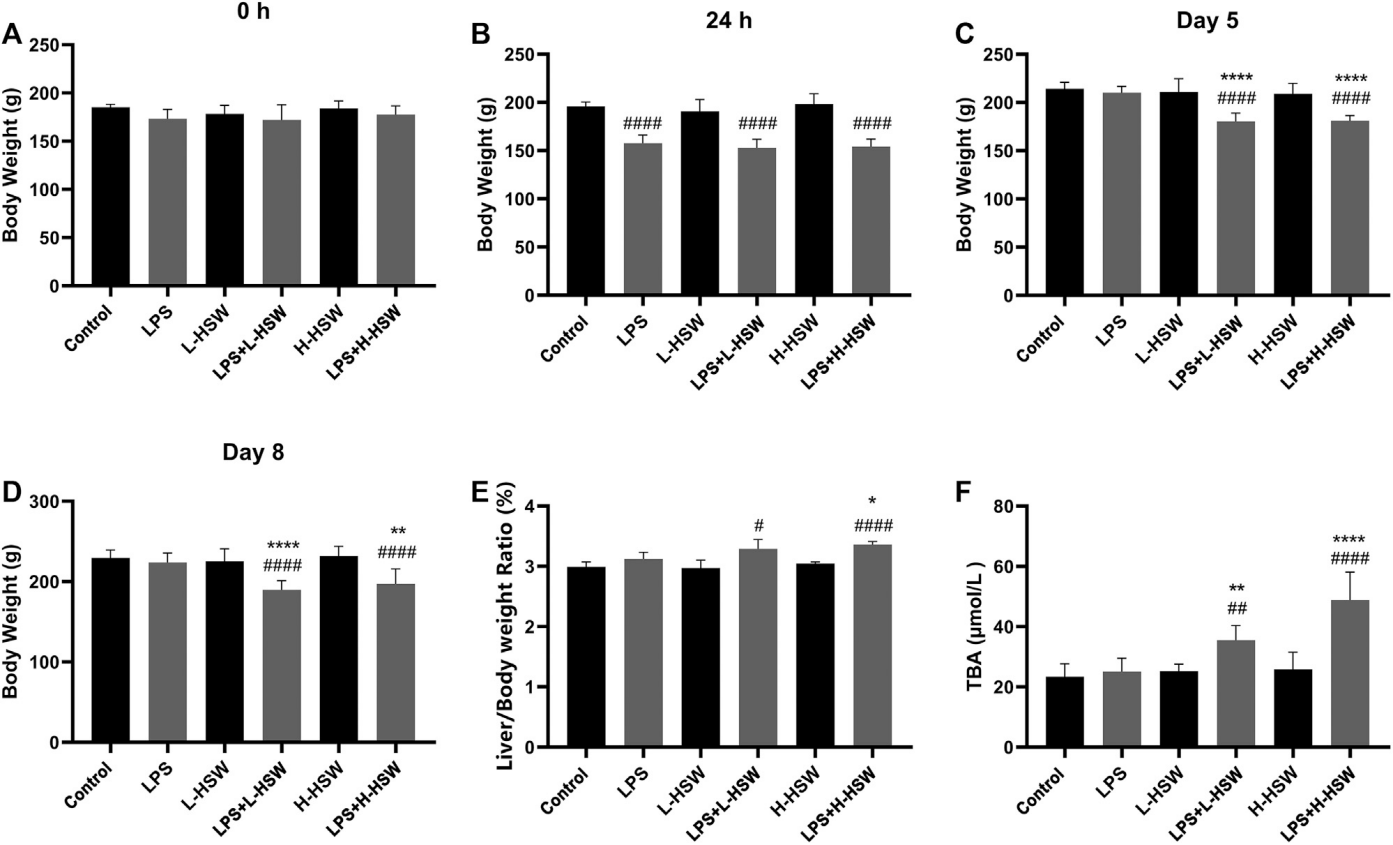
Histograms of body weights, liver/body ratios, and serum TBA levels. Six groups of rats were treated with saline (control), 2 mg/kg dose of LPS (LPS), 2 g/kg/day HSW (equivalent of raw herb, L-HSW), 10 g/kg/day HSW (equivalent of raw herb, H-HSW), 2 mg/kg LPS plus 2 g/kg/day HSW (LPS + L-HSW), and 2 mg/kg LPS plus 10 g/kg/day HSW (LPS + H-HSW), respectively. Body weights at 0 h **(A)**, 24 h **(B)**, day 5 **(C)**, and day 8 **(D)** as well as the liver/body ratio at day 8 **(E)** were measured; serum TBA levels **(F)** were determined with corresponding kits. Each bar represents the mean ± standard error (*n* = 6). ^#^ *p* < 0.05, ^##^ *p* < 0.01, ^###^ *p* < 0.001, and ^####^ *p* < 0.0001 comparing with control group. ^*^ *p* < 0.05, ^**^ *p* < 0.01, ^***^ *p* < 0.001, and ^****^ *p* < 0.0001 comparing with LPS group.

Both the rats treated with LPS + L-HSW and LPS + H-HSW showed significant body loss from the fifth day to the final day (*p* < 0.0001 *vs*. control and *p* < 0.0001 *vs.* LPS groups on the fifth day; *p* < 0.0001 *vs*. control group and *p* < 0.001 *vs.* LPS groups on the eigth day), while those rats treated solely with L-HSW or H-HSW did not exhibit the obvious body change (Figures 1A–D). The LPS group showed much lower body weight on the second days (*p* < 0.0001 *vs*. control), yet its gradual recovery on the rest days was observed by comparison with the control group. The ratio of liver to body weight on the final day was further calculated. Significant higher ratios of liver to body weight were observed in both LPS + H-HSW and LPS + L-HSW groups than control group (*p* < 0.05 L + HSW *vs.* control, *p* < 0.0001 H + HSW *vs.* control), while other groups did not showed any remarkable difference as compared with control group (Figure 1E), indicating that co-treatment of HSW and LPS induced moderate liver swelling in rats.

The serum TBA levels were increased in L + HSW (35.51 ± 4.84 μmol/L, *p* < 0.01 *vs*. control and *vs.* LPS group) and H + HSW groups (48.86 ± 9.21 μmol/L, *p* < 0.0001 *vs*. control and *vs.* LPS group) in a dose dependent manner (Figure 1F), while being treated with LPS or HSW solely did not vary the TBA levels (*p* > 0.05).

**FIGURE 2:**
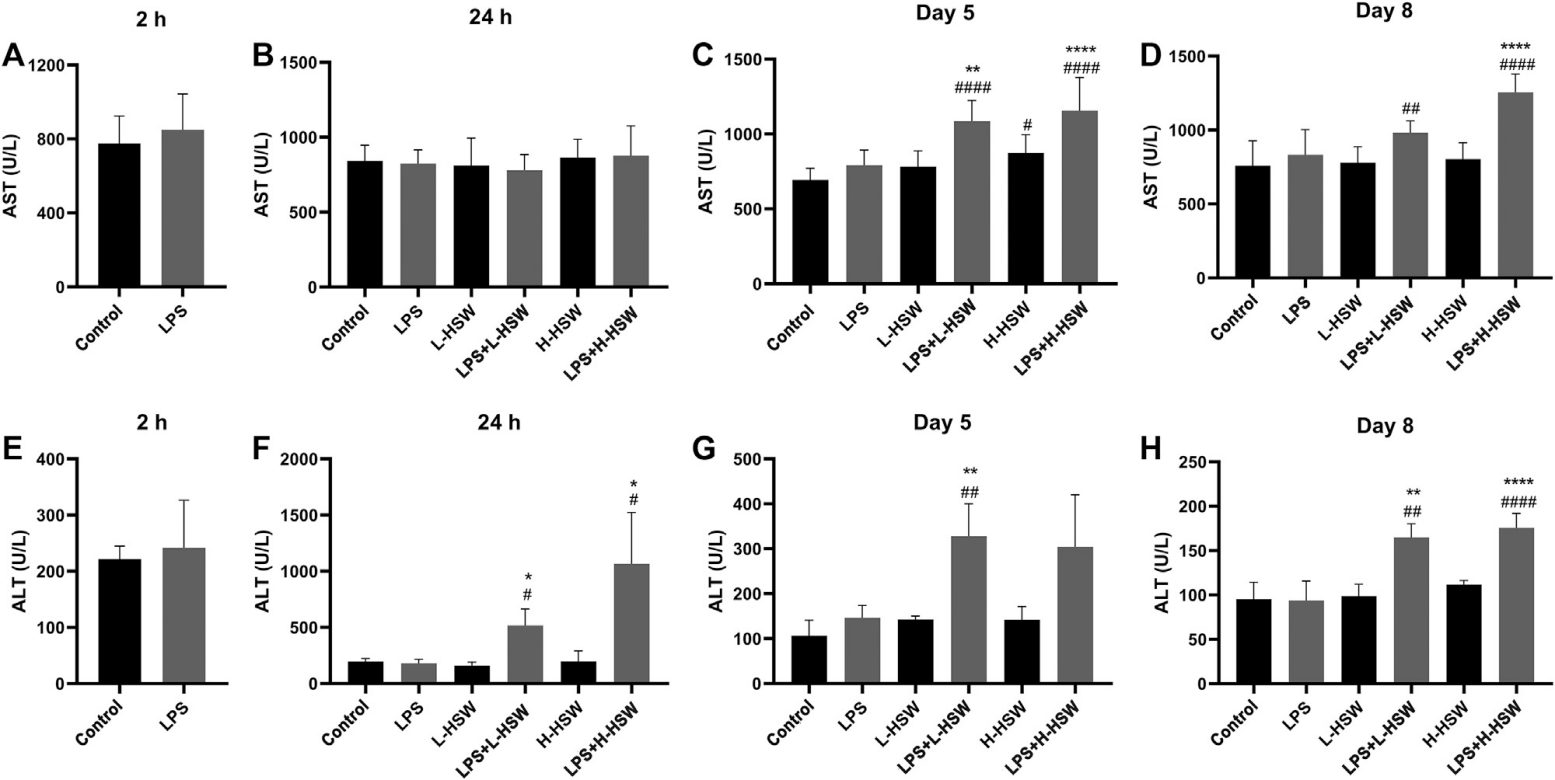
Histograms of serum ALT and AST levels. Serum AST and ALT levels were determined with corresponding assay kits. Groups of rats were treated with saline (control), 2 mg/kg dose of LPS (LPS), 2 g/kg/day HSW (equivalent of raw herb, L-HSW), 10 g/kg/day HSW (equivalent of raw herb, H-HSW), 2 mg/kg LPS plus 2 g/kg/day HSW (LPS + L-HSW), and 2 mg/kg LPS plus 10 g/kg/day HSW (LPS + H-HSW), respectively. **(A)** to **(D)** showed the AST levels at 2 h, 24 h, day 5 and day 8, respectively; **(E)** to **(H)** showed the ALT levels at 2 h, 24 h, day 5 and day 8, respectively. Each bar represents the mean ± standard error (*n* = 6). ^#^ *p* < 0.05, ^##^ *p* < 0.01, ^###^ *p* < 0.001, and ^####^ *p* < 0.0001 comparing with control group. ^*^ *p* < 0.05, ^**^ *p* < 0.01, ^***^ *p* < 0.001, and ^****^ *p* < 0.0001 comparing with LPS group.

**FIGURE 3:**
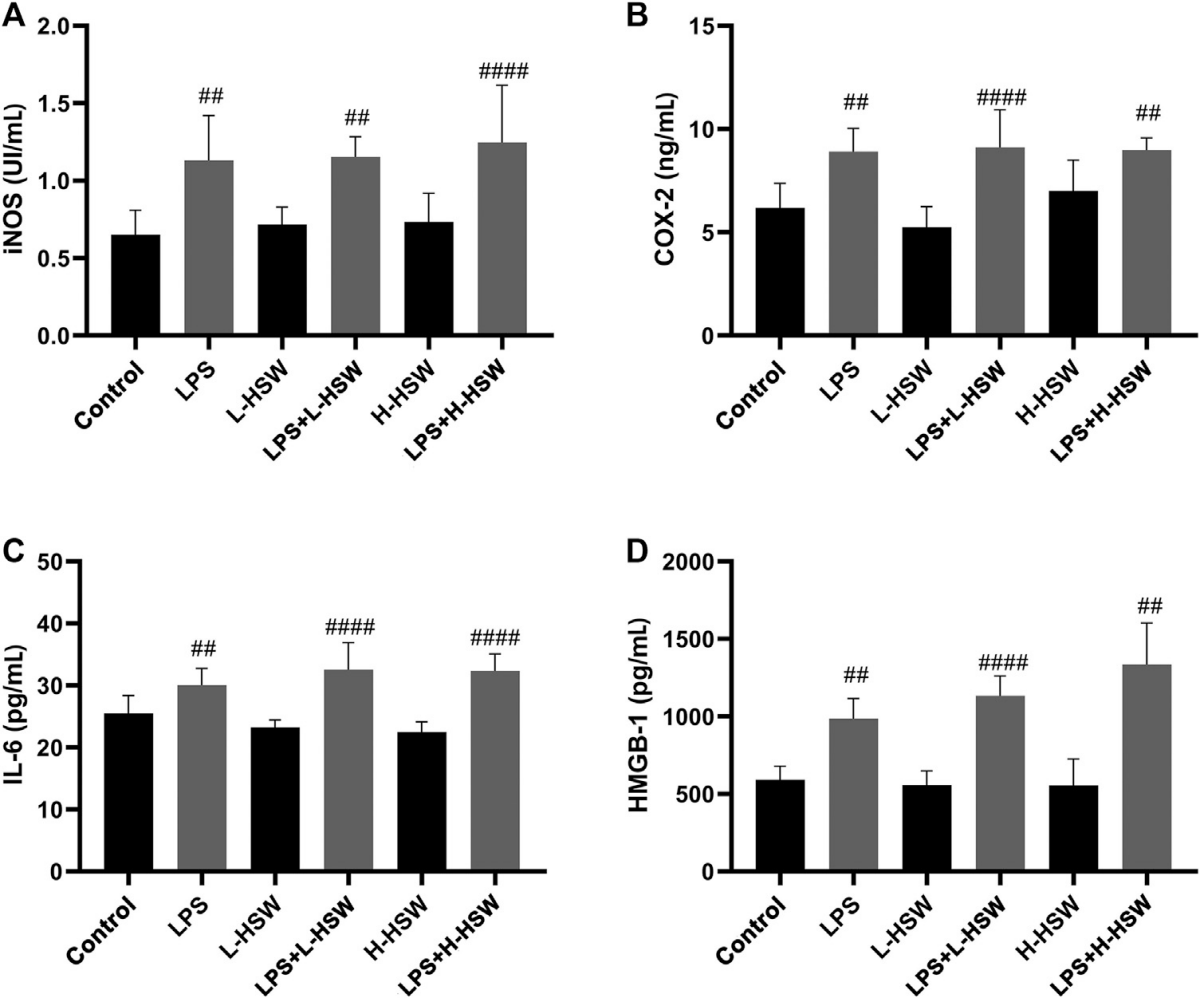
Histograms of levels of iNOS **(A)**, COX-2 **(B)**, IL-6 **(C)** and HMGB-1 **(D)**, which were determined by using the corresponding ELISA assay kits. Each bar represents the mean ± standard error (*n* = 6). ^#^ *p* < 0.05, ^##^ *p* < 0.01, ^###^ *p* < 0.001, and ^####^ *p* < 0.0001 comparing with control group. Comparing with LPS group, LPS + L-HSW and LPS + H-HSW groups did not showed significant changes.

Serum ALT and AST levels showed no significant changes in the groups of LPS, L-HSW, or H-HSW during the whole experimental period (*p* > 0.05) except that AST was slightly increased in the H-HSW group on the fifth day (*p* < 0.05), indicating that tail vein injection of LPS or oral administration of HSW at current dosages did not affect the liver function. Co-treatment of LPS and HSW, however, caused ALT increasing on the second, fifth, and eighth days and AST slightly increasing at the fifth and eighth days by comparison with LPS and control groups (Figure 2). Although previous study reported that some plasma chemokines and pro-inflammatory cytokines were induced by HSW (Tu et al., 2019), the four inflammatory cytokines of iNOS, IL-6, COX-2 and HMGB-1 did not showed significant changes in the LPS + HSW groups (*p* > 0.05 *vs.* LPS group, Figure 3) in present work.

**FIGURE 4:**
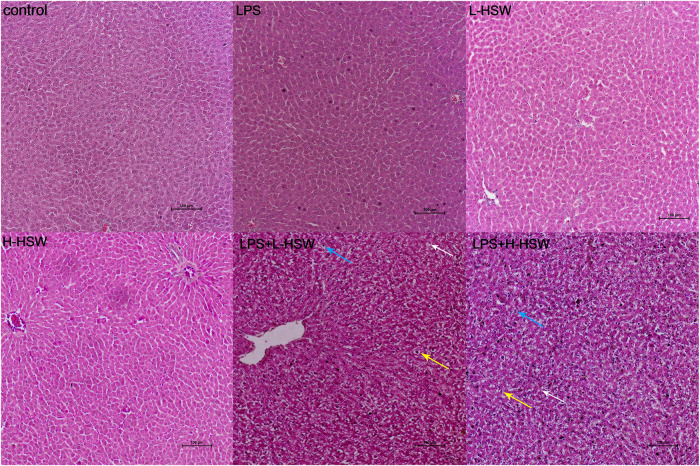
Representative histopathological microphotographs of rat liver. Rats were treated with saline (control), 2 mg/kg dose of LPS (LPS), 2 g/kg/day HSW (equivalent of raw herb, L-HSW), 10 g/kg/day HSW (equivalent of raw herb, H-HSW), 2 mg/kg LPS plus 2 g/kg/day HSW (equivalent of raw herb, LPS + L-HSW), and 2 mg/kg LPS plus 10 g/kg/day HSW (equivalent of raw herb, LPS + H-HSW). Examples of the histopathological abnormity of inflammatory cell infiltration, slight fat droplet, and visible swelling were indicated by blue, white, and yellow arrows, respectively. (H&E stained, 100 *μ*m indicated in the pictures).

The morphological feature of liver tissue is considered as a direct and critical evidence for the diagnosis of liver damage. Liver histologic examination on the eigth day revealed that the coalescent of LPS and HSW (group E and F) led to evident liver injury. As shown in Figure 4, co-treatment with LPS and HSW (group E and F) caused significant histopathological changes, including significant vacuolation in the cytoplasm, hepatic steatosis, pyknotic nucleus, karyorrhexis, and even focal necrosis. In addition, inflammatory cell infiltration, slight empty bubble fat droplet, and visible swelling were also observed in the two groups (Figure 4), whereas solely treatment with LPS or HSW showed regular liver histology comparing with control group. In conclusion, all of the changes indicated that the model of idiosyncratic liver injury rats induced by HSW was successfully built, and a potential connection between the liver injury and lipid remodeling induced by HSW was implicated as a result of the hepatic steatosis during the construction of this model.

### Untargeted Lipidomics Analysis of Liver in IDILI Rats Caused by HSW

**FIGURE 5:**
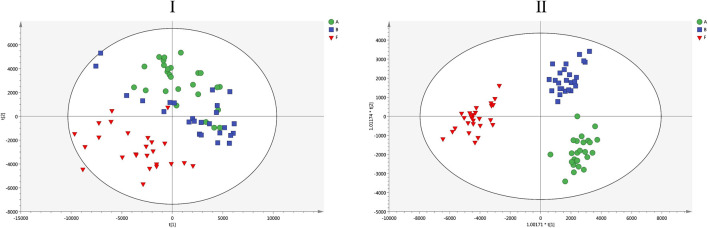
The score plots of PCA (I) and OPLS-DA (II) models for the untargeted lipidomics analysis. I: R2X and Q2 values of the PCA model are 0.741 and 0.544, respectively; II: R2Y and Q2 values of the OPLS-DA model are 0.985 and 0.963, respectively. Green circles, blue squares and red inverted triangles denote groups control **(A)**, LPS **(B)** and LPS + H-HSW **(F)**, respectively.

An untargeted lipidomics analysis of liver samples as conducted based on an UHPLC-QE-Orbitrap-MS. Livers from control, LPS, and LPS + H-HSW groups were selected for lipidomics analysis by an optimized UHPLC-QE-Orbitrap-MS method. Examples of total ion chromatograms (TIC) of group control, LPS, and LPS + H-HSW in negative and positive modes were shown in Supplementary Figure S2. The mega MS data of negative mode were imported into R using XCMS package for peak detection, alignment, correspondence, and normalization. A data matrix containing more than 2000 features was then led into SIMCA-P software for further PCA and OPLS-DA analysis. The PCA score plot (Figure 5I) demonstrated that the LPS + HSW samples could be distinguished from two other groups, while control group (A) and LPS group (B) were clustered together. The dataset was then applied to a supervised OPLS-DA analysis. Those liver samples of rats co-treated with LPS and HSW were clearly discriminated from samples of control and LPS groups. The quality of both two models were assessed by calculating the R2 and Q2 values. The R2X and Q2 values for the PCA model are 0.741 and 0.544, respectively, and the R2Y and Q2 values for the OPLS-DA model are 0.985 and 0.963, respectively. These large values indicated the good abilities of fitness and of prediction of the two models (Zhang et al., 2018). The permutations test was applied 200 times to further assess the predictability of the OPLS-DA model (Supplementary Figure S3). The validity of the original model was indicated as having lower Q2 and R2 values to the left compared to the original points (on the right) as well as intersection of the vertical axis (on the left) by the regression line of the Q2 points below zero in the permutations test.

### Identification of Lipid in Liver Samples

For identification of lipid in liver samples, each high-resolution MS peak of the LC-MS chromatograms extracted by Compound Discoverer™ were preliminarily screened and classified into lipid subspecies based on their distinct fragmentation patterns (Narváez-Rivas et al., 2017), and they were further confirmed by comparing the accurate mass determination and given molecular formula with lipid database on Lipidmap (http://www.lipidmaps.org/). Ultra-high accurate precursor ions determined with mass errors less than 1 ppm, mainly including deprotonated and formyl-adducted ions, coupled with the ^13^C isotope ratio pattern and nitrogen rule filtering were used for generation of exact molecular formula of each lipid. Subspecies of the identified lipids and their characterized fragments are summarized in Table 1. In most cases, PE, lysophosphatidylethanolamine (LPE), phosphatidylinositol (PI), lysophosphatidylinositol (LPI), phosphatidylserine (PS), and phosphoglycerols (PG) tend to be generated deprotonated ions, while formyl-adducted ion ([M + HCOO]) is more likely to be produced for PC, lysophosphatidylcholine (LPC), sphingomyelin (SM), and ceramide (Cer) in QE Orbitrap MS. Generally, the fragmentation of all of the phospholipids and lyso-phospholipids were characterized by loss of fatty acid (FA) residue at sn-1 or sn-2 of the glycerol, which were used to conform the acyl chains. For the alkyl or alkenyl substituted glycerol phosphates at sn-1, only [FA-H] fragments at sn-2 were detected. Take PE (20:4/18:0) as an example. It generated deprotonated ion at *m/z* 766.5396 (C_43_H_78_NO_8_P, 0.50 ppm), and then fragmented into two prominent ions in MS/MS spectrum 303.2323 (C_20_H_31_O_2_) and 283.2639 (C_18_H_35_O_2_), denoting two fatty acyl residue C20:4 and C18:0, respectively. Two species of sphingolipids (SM and Cer) detected in present study have a fatty amide instead of a fatty acyl ester group, which makes the cleavage of this bond more difficult. The main fragments of these lipids were characterized by neutral losses of CH_2_ and CH_2_O for SM at the choline residue and Cer at the sphingosine residue, respectively (Narváez-Rivas et al., 2017). Based on the retention time, exact mass determination of quasi-molecular and characteristic fragment ions, as well as the distinct neutral losses for each species, more than 202 lipid metabolites were detected in each liver sample, including PC, LPC, PE, LPE, PI, LPI, PG, PS, Cer, and SM. Information on retention time, quasi-molecular ion, mass error, and characteristic fragment ion of 202 lipids is listed in Table 2.

**TABLE 1:** The summarized LC-MS characters of the lipid species in rat liver.

Lipid species		Adducts	Total number	Characteristic fragment	Neutral loss	RT range
LPC		[M + HCOO]	25	[FA-H]	GPC-H_2_O	6.60–10.10
PC	PC	[M + HCOO]	5	[sn-1 FA-H]	sn-2-acyl GPC-H_2_O	13.70–16.00
				[sn-2 FA-H]	sn-1-acyl GPC-H_2_O	
	PC-O	[M + HCOO]	41	[FA-H]	sn-1-alkyl GPC-H_2_O	14.25–15.66
	PC-P	[M + HCOO]	1	[sn-2 FA-H]	sn-1-alkenyl GPC-H_2_O	15.72
LPE		[M-H]	29	[FA-H]	GPE-H_2_O	7.08–10.60
PE	PE	[M-H]	20	[sn-1 FA-H]	sn-1-acyl GPE-H_2_O	14.10–16.25
				[sn-2 FA-H]	sn-2-acyl GPE-H_2_O	
	PE-P	[M-H]	16	[sn-2 FA-H]	sn-1-alkenyl GPE-H_2_O	14.70–16.25
LPI		[M-H]	8	[FA-H]	GPI- H_2_O	6.60–8.91
PI		[M-H]	15	[sn-1 FA-H]	sn-2-acyl GPI-H_2_O	13.06–14.85
				[sn-2 FA-H]	sn-1-acyl GPI-H_2_O	
PG		[M-H]	11	[sn-1 FA-H]	sn-2-acyl GPG -H_2_O	13.06–14.05
				[sn-2 FA-H]	sn-1-acyl GPG-H_2_O	
PS		[M-H]	8	[sn-1 FA-H]	sn-2-acyl GPS-H_2_O	13.74–14.96
				[sn-2 FA-H]	sn-1-acyl GPS-H_2_O	
SM		[M + HCOO]	16	[M-CH_2_-H]	CH_2_	13.85–17.04
		[M-H]				
Cer		[M + HCOO]	7	[M-CH_2_O-H]	CH_2_O	15.20–17.45

FA: fatty acid; PC-O: alkyl, acylglycerophosphocholine; PC-P: alkenyl, acylglycerophosphocholine; GPE: glycero-3-phosphoethanolamine; PE-P: alkenyl, acyl glycerophosphoethanolamine GPC: glycero-3-phosphocholine; GPG: glycero-3-phosphoglycerol; GPI: glycero-3-phosphoinositol; GPS: glycero-3-phospho-L-serine.

**TABLE 2:** The detected and tentatively identified lipid molecules in the rat liver.

No	***R***_t_	m/z	Mass errors (ppm)	Molecular formula	Characteristic Fragment ions	Identification
1.	6.62	643.2894 [M − H]^−^	0.80	C_31_H_49_O_12_P	327.2317, 241.0111	LPI (22:6)
2.	6.65	512.2990 [M + HCOO]^−^	−0.77	C_22_H_46_NO_7_P	227.2005, 452.2768, 242.07957	LPC (14:0)
3.	6.70	619.2891 [M − H]^−^	0.35	C_29_H_49_O_12_P	303.2322, 315.0479, 241.0110	LPI (20:4)
4.	6.76	586.3153 [M + HCOO]^−^	0.44	C_28_H_48_NO_7_P	301.2156, 257.22681, 242.0790	LPC (20:5)
5.	6.82	643.2894 [M − H]^−^	0.80	C_31_H_49_O_12_P	327.2317, 241.0111	LPI (22:6)
6.	6.90	562.3150 [M + HCOO]^−^	−0.07	C_26_H_48_NO_7_P	277.2168,388.9909, 242.0794	LPC (18:3)
7.	6.97	595.2890 [M − H]^−^	0.19	C_27_H_49_O_12_P	279.2324, 315.0479, 241.0111	LPI (18:4)
8.	7.04	595.2890 [M − H]^−^	0.19	C_27_H_49_O_12_P	279.2324, 315.0479, 241.0111	LPI (18:4)
9.	7.05	619.2891 [M − H]^−^	0.35	C_29_H_49_O_12_P	303.2322, 315.0479, 241.0110	LPI (20:4)
10.	7.06	498.2625 [M − H]^−^	−0.23	C_25_H_42_NO_7_P	301.2166, 257.2271	LPE (20:5)
11.	7.08	498.2625 [M − H]^−^	−0.23	C_25_H_42_NO_7_P	301.2166,257.2271	LPE (20:5)
12.	7.09	512.2990 [M + HCOO]^−^	−0.77	C_22_H_46_NO_7_P	227.2005, 452.2768, 242.07957	LPC (14:0)
13.	7.16	450.2625 [M − H]^−^	−0.25	C_21_H_42_NO_7_P	253.2168, 419.1788, 289.1805	LPE (16:1)
14.	7.17	538.3149 [M + HCOO]^−^	−0.26	C_24_H_48_NO_7_P	253.2170, 478.2946, 242.0792	LPC (16:1)
15.	7.43	612.3307 [M + HCOO]^−^	0.01	C_30_H_50_NO_7_P	327.2319, 242.0789	LPC (22:6)
16.	7.44	450.2625 [M − H]^−^	−0.25	C_21_H_42_NO_7_P	253.2168, 419.1788, 289.1805	LPE (16:1)
17.	7.48	538.3149 [M + HCOO]^−^	−0.26	C_24_H_48_NO_7_P	253.2170, 478.2946, 242.0792	LPC (16:1)
18.	7.55	504.3091 [M − H]^−^	−0.30	C_25_H_48_NO_7_P	307.2635, 279.2333, 242.079	LPE (20:2)
19.	7.56	500.2785 [M − H]^−^	0.48	C_25_H_44_NO_7_P	303.2323, 259.2428, 214.0473	LPE (20:4)
20.	7.57	588.3308 [M + HCOO]^−^	0.18	C_28_H_50_NO_7_P	303.2322, 528.3073, 259.2427, 242.0790	LPC (20:4)
21.	7.60	524.2778 [M − H]^−^	−0.88	C_27_H_44_NO_7_P	327.2319, 283.2427, 249.1855, 229.1947	LPE (22:6)
22.	7.61	564.3306 [M + HCOO]^−^	−0.16	C_26_H_52_NO_7_P	504.3120, 279.2325, 242.0790, 224.0684	LPC (18:2)
23.	7.63	612.3307 [M + HCOO]^−^	0.01	C_30_H_50_NO_7_P	327.2319, 242.0789	LPC (22:6)
24.	7.68	476.2779 [M − H]^−^	−0.76	C_23_H_44_NO_7_P	279.2327, 214.0473	LPE (18:2)
25.	7.73	524.2778 [M − H]^−^	−0.88	C_27_H_44_NO_7_P	327.2319, 283.2427, 249.1855, 229.1947	LPE (22:6)
26.	7.74	476.2779 [M − H]^−^	−0.76	C_23_H_44_NO_7_P	279.2327, 214.0473	LPE (18:2)
27.	7.74	526.3145 [M + HCOO]^−^	−0.44	C_23_H_48_NO_7_P	241.2165, 328.2353, 284.2460	LPC (15:0)
28.	7.78	588.3308 [M + HCOO]^−^	0.18	C_28_H_50_NO_7_P	303.2322, 528.3073, 259.2427, 242.0790	LPC (20:4)
29.	7.82	614.3463 [M + HCOO]^−^	−0.07	C_30_H_52_NO_7_P	329.2475, 285.2583, 554.3242	LPC (22:5)
30.	7.84	564.3306 [M + HCOO]^−^	−0.16	C_26_H_52_NO_7_P	504.3120, 279.2325, 242.0790, 224.0684	LPC (18:2)
31.	7.84	504.3091 [M − H]^−^	−0.30	C_25_H_48_NO_7_P	307.2635, 279.2333, 242.079	LPE (20:2)
32.	7.87	500.2785 [M − H]^−^	0.48	C_25_H_44_NO_7_P	303.2323, 259.2428, 214.0473	LPE (20:4)
33.	7.91	526.2939 [M − H]^−^	−0.02	C_27_H_46_NO_7_P	329.2476, 285.2582, 214.0473	LPE (22:5)
34.	7.91	526.2939 [M − H]^−^	−0.02	C_27_H_46_NO_7_P	329.2476, 285.2582, 214.0473	LPE (22:5)
35.	8.05	614.3463 [M + HCOO]^−^	−0.07	C_30_H_52_NO_7_P	329.2475, 285.2583, 554.3242	LPC (22:5)
36.	8.20	540.3312 [M + HCOO]^−^	0.94	C_24_H_50_NO_7_P	255.2326, 480.3085, 242.0790, 224.0687	LPC (16:0)
37.	8.25	452.2783 [M − H]^−^	0.08	C_21_H_44_NO_7_P	255.2325, 383.2892, 214.0472	LPE (16:0)
38.	8.36	480.3095 [M − H]^−^	−0.13	C_23_H_48_NO_7_P	283.2641, 255.2323, 224.0681	LPE (18:0)
39.	8.42	526.2939 [M − H]^−^	−0.02	C_27_H_46_NO_7_P	329.2476, 285.2582, 214.0473	LPE (22:5)
40.	8.47	566.3464 [M + HCOO]^−^	0.10	C_26_H_50_NO_7_P	281.2483, 506.3242, 242.0791	LPC (18:1)
41.	8.48	540.3312 [M + HCOO]^−^	0.94	C_24_H_50_NO_7_P	255.2326, 480.3085, 242.0790, 224.0687	LPC (16:0)
42.	8.48	478.2936 [M − H]^−^	−0.65	C_23_H_46_NO_7_P	281.2485, 255.2331, 214.0473	LPE (18:1)
43.	8.54	452.2783 [M − H]^−^	0.08	C_21_H_44_NO_7_P	255.2325, 383.2892, 214.0472	LPE (16:0)
44.	8.57	599.3207 [M − H]^−^	0.86	C_27_H_53_O_12_P	283.2638, 315.0479, 241.0110	LPI (18:0)
45.	8.75	566.3464 [M + HCOO]^−^	0.10	C_26_H_50_NO_7_P	281.2483, 506.3242, 242.0791	LPC (18:1)
46.	8.77	592.3629 [M + HCOO]^−^	0.90	C_28_H_54_NO_7_P	307.2635, 532.3385, 357.0862, 242.0788	LPC (20:2)
47.	8.84	478.2936 [M − H]^−^	−0.65	C_23_H_46_NO_7_P	281.2485, 255.2331, 214.0473	LPE (18:1)
48.	8.91	599.3207 [M − H]^−^	0.86	C_27_H_53_O_12_P	283.2638, 315.0479, 241.0110	LPI (18:0)
49.	9.04	478.2936 [M − H]^−^	−0.65	C_23_H_46_NO_7_P	281.2485, 255.2331, 214.0473	LPE (18:1)
50.	9.04	592.3629 [M + HCOO]^−^	0.90	C_28_H_54_NO_7_P	307.2635, 532.3385, 357.0862, 242.0788	LPC (20:2)
51.	9.16	554.3463 [M + HCOO]^−^	−0.07	C_25_H_52_NO_7_P	269.2484, 494.3245, 242.0791, 224.0683	LPC (17:0)
52.	9.26	466.2937 [M − H]^−^	0.21	C_22_H_46_NO_7_P	269.2484, 196.0367	LPE (17:0)
53.	9.48	508.3403 [M − H]^−^	−1.10	C_25_H_52_NO_7_P	283.2646, 242.0791, 224.0683	LPE (20:0)
54.	9.56	568.3622 [M + HCOO]^−^	0.37	C_26_H_54_NO_7_P	283.2640, 508.3399, 242.0790, 224.0680	LPC (18:0)
55.	9.70	480.3095 [M − H]^−^	−0.13	C_23_H_48_NO_7_P	283.2641, 255.2323, 224.0681	LPE (18:0)
56.	9.84	568.3624 [M + HCOO]^−^	0.78	C_26_H_54_NO_7_P	283.2640, 508.3399, 242.0790, 224.0680	LPC (18:0)
57.	9.86	508.3403 [M − H]^−^	−1.10	C_25_H_52_NO_7_P	283.2646, 242.0791, 224.0683	LPE (20:0)
58.	10.04	594.3782 [M + HCOO]^−^	0.93	C_28_H_56_NO_7_P	309.2793, 534.3554, 357.0879, 224.0682	LPC (20:1)
59.	10.07	506.3248 [M − H]^−^	−0.80	C_25_H_50_NO_7_P	309.2793, 281.2482, 214.0476	LPE (20:1)
60.	10.10	480.3095 [M − H]^−^	−0.13	C_23_H_48_NO_7_P	283.2641, 255.2323, 224.0681	LPE (18:0)
61.	10.33	494.3251 [M − H]^−^	−0.23	C_24_H_50_NO_7_P	297.2795, 214.0475	LPE (19:0)
62.	10.59	494.3251 [M − H]^−^	−0.23	C_24_H_50_NO_7_P	297.2793, 405.2762, 214.0476	LPE (19:0)
63.	12.23	865.5023 [M − H]^−^	−0.24	C_50_H_75_O_10_P	327.2321, 355.9512, 283.2428	PG (22:6/22:6)
64.	12.55	817.5028 [M − H]^−^	0.36	C_46_H_75_O_10_P	327.2321, 279.2328, 463.3472	PG (22:6/18:2)
65.	12.73	793.5029 [M − H]^−^	0.49	C_44_H_75_O_10_P	303.2323, 279.2328	PG (20:4/18:2)
66.	12.82	769.5028 [M − H]^−^	0.38	C_42_H_75_O_10_P	279.2328, 397.9163, 223.1688	PG (18:2/18:2)
67.	13.06	881.5187 [M − H]^−^	0.17	C_47_H_79_O_13_P	303.2324, 279.2326, 241.0112	PI (38:6)
68.	13.14	819.5188 [M − H]^−^	−0.47	C_46_H_77_O_10_P	327.2319, 281.2484	PG (22:6/18:1)
69.	13.29	793.5029 [M − H]^−^	0.49	C_44_H_75_O_10_P	303.2323, 279.2328, 255.2325	PG (20:4/18:2)
70.	13.37	769.5028 [M − H]^−^	0.38	C_42_H_75_O_10_P	279.2328, 397.9163, 223.1688	PG (18:2/18:2)
71.	13.50	881.5187 [M − H]^−^	0.17	C_47_H_79_O_13_P	327.2321, 255.2326, 241.0112	PI (22:6/16:0)
72.	13.66	857.5184 [M − H]^−^	−0.18	C_45_H_79_O_13_P	303.2322, 255.2326, 241.0112	PI (16:0/20:4)
73.	13.68	833.5190 [M − H]^−^	0.54	C_43_H_79_O_13_P	279.2328, 255.2327, 241.0112	PI (18:2/16:0)−H−833
74.	13.74	806.4974 [M − H]^−^	−0.43	C_44_H_74_NO_10_P	327.2319, 255.2326	PS (22:6/16:0)
75.	13.76	793.5029 [M − H]^−^	0.49	C_44_H_75_O_10_P	303.2324, 255.2326	PG (22:6/16:0)
76.	13.76	769.5028 [M − H]^−^	0.38	C_42_H_75_O_10_P	303.2323, 255.2326	PG (20:4/16:0)
77.	13.78	883.5344 [M − H]^−^	0.22	C_47_H_81_O_13_P	303.2324, 281.248,4,241.0112	PI (20:4/18:1)
78.	13.79	824.5452 [M + HCOO]^−^	−0.34	C_44_H_78_NO_8_P	303.2324, 253.2169, 224.0684	PC (20:4/16:1)
79.	13.83	824.5452 [M + HCOO]^−^	0.80	C_44_H_78_NO_8_P	279.2328, 502.2956	PC (18:3/18:2)
80.	13.84	798.5292 [M + HCOO]^−^	0.45	C_42_H_76_NO_8_P	303.2324, 227.2006, 452.2783	PC (14:0/20:4)
81.	13.85	745.5502 [M + HCOO]^−^	0.07	C_39_H_77_N_2_O_6_P	279.2327, 255.2325	SM (18:2/16:0)
82.	13.90	793.5029 [M − H]^−^	0.49	C_44_H_75_O_10_P	303.2324, 255.2326	PG (22:6/16:0)
83.	13.92	782.4980 [M − H]^−^	0.24	C_42_H_74_NO_10_P	303.2323, 255.2326	PS (20:4/16:0)
84.	13.92	883.5344 [M − H]^−^	0.22	C_47_H_81_O_13_P	301.2166, 283.2639, 241.0111	PI (20:5/18:0)
85.	13.96	898.5605 [M + HCOO]^−^	0.45	C_50_H_80_NO_8_P	327.2319, 303.2324	PC (20:4/22:6)
86.	13.98	871.5341 [M − H]^−^	−0.10	C_46_H_81_O_13_P	303.2324, 269.2484, 241.0111	PI (20:4/17:0)
87.	13.99	733.5499 [M + HCOO]^−^	−0.23	C_38_H_77_N_2_O_6_P	281.2483, 673.5270, 241.2165	SM (d18:1/15:0)
88.	14.04	769.5028 [M − H]^−^	0.29	C_42_H_75_O_10_P	303.2323, 255.2325	PG (20:4/16:0)
89.	14.07	874.5605 [M + HCOO]^−^	0.14	C_48_H_8_NO_8_P	327.2320, 279.2328, 224.0680	PC (20:4/18:2)
90.	14.08	774.5294 [M + HCOO]^−^	0.65	C_40_H_76_NO_8_P	279.2328, 227.2007, 452.2775	PC (18:2/14:0)
91.	14.11	800.5445 [M + HCOO]^−^	0.10	C_42_H_78_NO_8_P	279.2328, 253.2168, 224.0680	PC (18:2/16:1)
92.	14.11	824.5452 [M + HCOO]^−^	0.49	C_44_H_78_NO_8_P	303.2323, 478.2934	PC (20:4/18:1)
93.	14.13	736.4925 [M − H]^−^	0.53	C_41_H_72_NO_8_P	279.2326	PE (18:3/18:2)
94.	14.16	850.5606 [M + HCOO]^−^	0.24	C_46_H_80_NO_8_P	303.2323, 279.2327	PC (20:4/18:2)
95.	14.17	836.5450 [M − H]^−^	0.29	C_46_H_80_NO_10_P	327.2319, 283.2421, 241.2166	PS (22:6/18:0)
96.	14.20	859.5340 [M − H]^−^	−0.20	C_45_H_81_O_13_P	305.2480, 255.2327, 241.0113	PI (20:3/16:0)
97.	14.22	835.5342 [M − H]^−^	−0.01	C_43_H_81_O_13_P	281.2483, 255.2325, 241.0111	PI (18:1/16:0)
98.	14.24	909.5500 [M − H]^−^	0.14	C_49_H_83_O_13_P	327.2319, 283.2656, 419.2558, 241.0111	PI (22:6/18:0)
99.	14.25	826.5607 [M + HCOO]^−^	0.34	C_44_H_80_NO_8_P	301.2164, 255.2326, 504.3098, 224.0682	PC (O−16:0/20:4)
100.	14.27	736.4925 [M − H]^−^	0.22	C_41_H_72_NO_8_P	303.2324, 253.2169	PE (20:4/16:1)
101.	14.29	812.5449 [M + HCOO]^−^	0.19	C_43_H_78_NO_8_P	303.2324, 259.2427, 466.2927, 241.2166	PC (20:4/15:0)
102.	14.32	788.5452 [M + HCOO]^−^	0.49	C_41_H_78_NO_8_P	466.2934 279.2328 241.2166	PC (18:2/15:0)
103.	14.32	788.5451 [M − H]^−^	0.39	C_42_H_80_NO_10_P	283.2656, 281.2483, 466.2934, 241.2166	PS (18:1/18:0)
104.	14.35	876.5759 [M + HCOO]^−^	−0.10	C_48_H_82_NO_8_P	329.2477, 279.2327, 530.3259, 504.3091	PC (22:5/18:2)
105.	14.36	762.5081 [M − H]^−^	0.17	C_43_H_74_NO_8_P	303.2323, 279.2326	PE (20:4/18:2)
106.	14.39	800.5445 [M − H]^−^	0.10	C_42_H_78_NO_8_P	277.2171, 255.2326	PC (18:3/16:0)
107.	14.41	736.4925 [M − H]^−^	0.22	C_41_H_72_NO_8_P	301.2166, 255.2326	PE (20:5/16:0)
108.	14.42	885.5497 [M − H]^−^	−0.15	C_47_H_83_O_13_P	303.2324, 283.2639, 581.3086, 419.2560	PI (20:4/18:0)
109.	14.44	747.5661 [M + HCOO]^−^	−0.06	C_39_H_79_N_2_O_6_P	281.2482, 255.2325	SM (18:1/16:0)
110.	14.44	850.5601 [M + HCOO]^−^	−0.28	C_46_H_80_NO_8_P	303.2323, 279.2327	PC (20:4/18:2)
111.	14.45	911.5653 [M − H]^−^	−0.28	C_49_H_85_O_13_P	329.2477, 283.2640, 581.3077, 419.2560, 241.0112	PI (22:5/18:0)
112.	14.46	776.5446 [M − H]^−^	−0.11	C_40_H_78_NO_8_P	281.2483, 227.2007	PC (14:0/18:1)
113.	14.48	861.5497 [M − H]^−^	−0.54	C_45_H_83_O_13_P	283.2639, 279.2326, 581.3085, 419.2553, 241.0112	PI (18:2/18:0)
114.	14.49	834.5295 [M − H]^−^	0.13	C_46_H_78_NO_10_P	327.2320, 283.2643, 419.2559	PS (22:6/18:0)
115.	14.53	826.5607 [M + HCOO]^−^	0.08	C_44_H_80_NO_8_P	303.2324, 480.3083, 255.2327	PC (20:4/16:0)
116.	14.61	810.5294 [M − H]^−^	0.34	C_44_H_78_NO_10_P	303.2323, 283.2640, 437.2665, 419.2559	PS (20:4/18:0)
117.	14.62	876.5759 [M + HCOO]^−^	−0.09	C_48_H_82_NO_8_P	327.2321, 279.2327, 506.3261, 452.2776	PC (22:6/18:1)
118.	14.64	762.5081 [M − H]^−^	0.01	C_43_H_74_NO_8_P	327.2318, 452.2773, 255.2325	PE (22:6/16:0)
119.	14.68	802.5602 [M + HCOO]^−^	0.17	C_42_H_80_NO_8_P	279.2325, 255.2325, 480.3091, 224.0682	PC (18:2/16:0)
120.	14.70	887.5656 [M − H]^−^	−0.08	C_47_H_85_O_13_P	305.2480, 283.2640, 581.3088, 419.2560	PI (20:3/18:0)
121.	14.71	852.5761 [M + HCOO]^−^	−0.31	C_46_H_82_NO_8_P	303.2323, 281.2482, 224.0683	PC (20:4/18:1)
122.	14.71	788.5241 [M − H]^−^	−0.25	C_45_H_76_NO_8_P	327.2320, 281.2484, 478.2938	PE (22:6/18:1)
123.	14.75	786.5294 [M − H]^−^	0.61	C_42_H_78_NO_10_P	283.2640, 279.2326, 419.2559	PS (18:2/18:0)
124.	14.76	746.5133 [M − H]^−^	0.48	C_43_H_74_NO_7_P	303.2324, 442.2720, 280.2360, 259.2429	PE (P−16:0/22:6)
125.	14.80	828.5766 [M + HCOO]^−^	0.12	C_44_H_82_NO_8_P	279.2328, 255.2320, 224.0685	PC (18:2/18:1)
126.	14.84	887.5656 [M − H]^−^	0.10	C_47_H_85_O_13_P	305.2480, 283.2640, 581.3088, 419.2560	PI (20:3/18:0)
127.	14.86	738.5082 [M − H]^−^	0.27	C_41_H_74_NO_8_P	303.2324, 255.2326	PE (20:4/16:0)
128.	14.90	878.5918 [M + HCOO]^−^	0.23	C_48_H_84_NO_8_P	307.2636, 303.2324, 532.34011	PC (20:4/20:2)
129.	14.92	764.5235 [M − H]^−^	−0.07	C_43_H_76_NO_8_P	303.2324, 281.2483, 478.2932	PE (20:4/18:1)
130.	14.96	714.5085 [M − H]^−^	0.57	C_39_H_74_NO_8_P	279.2327, 255.2325	PE (18:2/16:0)
131.	14.96	808.5120 [M − H]^−^	−1.79	C_44_H_76_NO_10_P	303.2323, 281.2482, 478.2930	PS (20:4/18:1)
132.	14.98	840.5761 [M + HCOO]^−^	−0.09	C_45_H_82_NO_8_P	303.2323, 269.2484, 494.3237	PC (17:0/20:4)
133.	14.99	852.5761 [M + HCOO]^−^	0.09	C_46_H_82_NO_8_P	301.2167, 283.2640, 480.3083, 224.0682	PC (20:5/18:0)
134.	15.00	740.5239 [M − H]^−^	0.322	C_41_H_76_NO_8_P	279.2327, 478.2930	PE (18:2/18:1)
135.	15.00	810.5660 [M + HCOO]^−^	0.30	C_44_H_80_NO_7_P	303.2323, 283.2640, 464.3142	PC (O−16:0/20:5)
136.	15.01	866.5919 [M + HCOO]^−^	−0.19	C_47_H_84_NO_8_P	303.2324, 295.2637, 520.3400	PC (20:4/19:1)
137.	15.02	788.5241 [M − H]^−^	0.52	C_45_H_76_NO_8_P	327.2321, 283.2647, 505.2828, 419.2563	PE (22:7/18:0)
138.	15.03	746.5133 [M − H]^−^	0.30	C_43_H_74_NO_7_P	327.2322, 436.2824, 418.2716	PE (P−22:6/16:0)
139.	15.05	790.5395 [M − H]^−^	0.27	C_45_H_78_NO_8_P	329.2478, 281.2482	PE (22:5/18:1)
140.	15.06	778.5603 [M + HCOO]^−^	0.08	C_40_H_80_NO_8_P	255.2329, 480.3091, 224.0682	PC (16:0/16:0)
141.	15.06	764.5235 [M − H]^−^	−0.02	C_43_H_76_NO_8_P	301.2166, 283.2640, 480.3095	PE (20:5/18:0)
142.	15.07	812.5809 [M + HCOO]^−^	−0.13	C_44_H_82_NO_7_P	303.2324, 259.2428, 466.3292	PC (O−16:0/20:4)
143.	15.09	775.5970 [M + HCOO]^−^	0.19	C_41_H_83_N_2_O_6_P	281.2483, 269.2484	SM (d18:1/18:0)
144.	15.10	838.5968 [M + HCOO]^−^	0.28	C_46_H_84_NO_7_P	331.2633, 303.2323, 492.3454, 419.2560	PC (O−16:0/22:5)
145.	15.11	816.5766 [M + HCOO]^−^	0.59	C_43_H_82_NO_8_P	279.2327, 269.2484, 494.3242, 224.0684	PC (18:2/17:0)
146.	15.11	772.5288 [M − H]^−^	0.11	C_45_H_76_NO_7_P	303.2323, 259.2428, 436.2824, 418.2718	PE (P−18:1/22:6)
147.	15.18	878.5918 [M + HCOO]^−^	0.01	C_48_H_84_NO_8_P	508.3404 327.2320 283.2640 229.1951 224.0681 168.0417	PC (22:6/18:0)
148.	15.20	582.5104 [M + HCOO]^−^	0.45	C_34_H_67_NO_3_	281.2483, 255.2325	Cer (d18:1/16:0)
149.	15.23	804.5759 [M + HCOO]^−^	0.28	C_42_H_82_NO_8_P	281.2483, 255.232, 480.3109	PC (18:1/16:0)
150.	15.25	722.5130 [M − H]^−^	0.31	C_41_H_74_NO_7_P	303.2323, 436.2824, 418.2721	PE (P−16:0/20:4)
151.	15.28	854.5917 [M + HCOO]^−^	0.30	C_46_H_84_NO_8_P	303.2323, 283.2638, 508.3405	PC (20:4/18:0)
152.	15.30	790.5395 [M − H]^−^	0.56	C_45_H_78_NO_8_P	327.2318, 283.2661, 480.3081	PE (18:0/22:6)
153.	15.31	748.5288 [M − H]^−^	0.28	C_43_H_76_NO_7_P	329.2476, 303.2323, 462.2985, 444.2882	PE (P−16:0/22:5)
154.	15.32	880.6075 [M + HCOO]^−^	0.13	C_48_H_86_NO_8_P	283.2640, 534.3533, 508.3383	PC (22:5/18:0)
155.	15.36	853.6454 [M + HCOO]^−^	2.01	C_47_H_89_N_2_O_6_P	N.D.	SM (42:4)
156.	15.36	830.5921 [M + HCOO]^−^	0.60	C_44_H_84_NO_8_P	283.2638, 279.2328, 508.3400	PC (18:2/18:0)
157.	15.42	716.5238 [M − H]^−^	0.23	C_39_H_76_NO_8_P	281.2482, 255.2325	PE (18:1/16:0)
158.	15.45	748.5289 [M − H]^−^	0.05	C_43_H_76_NO_7_P	329.2476, 301.2165, 464.3139, 436.2825	PE (P−16:0/22:5)
159.	15.47	766.5396 [M − H]^−^	0.50	C_43_H_78_NO_8_P	303.2323, 283.2639, 480.3085	PE (20:4/18:0)
160.	15.49	742.5398 [M − H]^−^	0.93	C_41_H_78_NO_8_P	283.2637, 279.2327, 480.3091	PE (18:2/18:0)
161.	15.55	856.6069 [M + HCOO]^−^	−0.01	C_46_H_86_NO_8_P	305.2478, 283.2637, 508.3398	PC (20:3/18:0)
162.	15.55	854.5917 [M + HCOO]^−^	0.23	C_46_H_84_NO_8_P	303.2322, 283.2638, 508.3395	PC (20:4/18:0)
163.	15.59	880.6075 [M + HCOO]^−^	0.13	C_48_H_86_NO_8_P	329.2476, 283.2640, 508.3409, 224.0681	PC (22:5/18:0)
164.	15.61	868.6073 [M + HCOO]^−^	−0.29	C_47_H_86_NO_8_P	303.2322, 297.2792, 522.3557	PC (20:4/19:0)
165.	15.66	764.5815 [M + HCOO]^−^	0.76	C_40_H_82_NO_7_P	255.2328, 466.3296, 448.3191	PC (O−16:0/16:0)
166.	15.67	750.5444 [M − H]^−^	0.07	C_43_H_78_NO_7_P	303.2321, 464.3138, 436.2824, 418.2717	PE (P−18:0/20:4)
167.	15.68	776.5603 [M − H]^−^	−0.20	C_45_H_80_NO_7_P	331.2633, 283.2424, 462.2984, 444.2876	PE (P−18:1/22:4)
168.	15.68	844.6073 [M + HCOO]^−^	−0.15	C_45_H_86_NO_8_P	297.2792, 279.2327, 522.3567	PC (18:2/19:0)
169.	15.69	774.5443 [M − H]^−^	0.15	C_45_H_78_NO_7_P	327.2319, 283.2424, 464.3138, 446.3031	PE (P−18:0/22:6)
170.	15.72	792.5549 [M − H]^−^	−0.01	C_45_H_80_NO_8_P	329.2477, 283.2641, 480.3076, 255.2325	PE (22:5/18:0)
171.	15.72	790.5966 [M + HCOO]^−^	0.07	C_42_H_84_NO_7_P	283.2661, 281.2483, 255.2328, 492.3455	PC (P−16:0/18:0)
172.	15.73	882.6229 [M + HCOO]^−^	0.04	C_48_H_88_NO_8_P	331.2633, 283.2639, 508.3401	PC (22:4/18:0)
173.	15.78	832.6079 [M + HCOO]^−^	0.44	C_44_H_86_NO_8_P	283.2637, 281.2483, 508.3392	PC (18:1/18:0)
174.	15.81	750.5444 [M − H]^−^	−0.01	C_43_H_78_NO_7_P	303.2323, 259.2427, 464.3137, 446.3030	PE (P−16:0/22:4)
175.	15.82	776.5603 [M − H]^−^	−0.03	C_45_H_80_NO_7_P	303.2323, 285.2582, 464.3139, 446.3033	PE (P−18:1/22:4)
176.	15.84	806.5921 [M + HCOO]^−^	1.23	C_42_H_84_NO_8_P	283.2638, 255.2325, 745.6108	PC (16:0/18:0)
177.	15.86	803.6286 [M + HCOO]^−^	0.13	C_43_H_87_N_2_O_6_P	279.2328, 255.2325, 743.6052	SM (d18:1/20:0)
178.	15.87	882.6229 [M + HCOO]^−^	0.04	C_48_H_88_NO_8_P	331.2632, 283.2639, 259.2427	PC (22:4/18:0)
179.	15.88	829.6446 [M + HCOO]^−^	0.53	C_45_H_89_N_2_O_6_P	283.2639, 279.2326, 769.6205	SM (d18:0/22:2)
180.	15.91	744.5555 [M − H]^−^	0.73	C_41_H_80_NO_8_P	283.2638, 281.2484, 480.3101	PE (18:1/18:0)
181.	15.94	752.5601 [M − H]^−^	0.37	C_43_H_80_NO_7_P	305.2478, 464.3139, 446.3031	PE (P−18:0/20:3)
182.	15.96	855.6599 [M + HCOO]^−^	−0.15	C_47_H_91_N_2_O_6_P	303.2323, 283.2639, 795.6364	SM (42:3)
183.	15.96	776.5603 [M − H]^−^	0.20	C_45_H_80_NO_7_P	329.2477, 285.2583, 464.3138, 446.3030	PE (P−18:1/22:4)
184.	15.97	858.6231 [M + HCOO]^−^	0.60	C_46_H_88_NO_8_P	307.2636, 283.2639, 797.6429	PC (20:2/18:0)
185.	16.10	778.5760 [M − H]^−^	0.56	C_45_H_82_NO_7_P	331.2634,287.2739, 464.3138, 446.3035	PE (P−18:0/22:4)
186.	16.10	780.5912 [M − H]^−^	−1.12	C_45_H_84_NO_7_P	303.2322, 297.2792, 494.3253	PE (P−18:0/22:3)
187.	16.21	792.5549 [M − H]^−^	0.22	C_45_H_80_NO_8_P	283.2642, 255.2326, 494.3606	PE (22:5/18:0)
188.	16.21	843.6598 [M + HCOO]^−^	0.21	C_46_H_91_N_2_O_6_P	281.2483, 783.6366	SM (d18:1/19:1)
189.	16.24	778.5760 [M − H]^−^	0.16	C_45_H_82_NO_7_P	331.2633, 303.2323, 492.3455	PE (P−18:0/22:4)
190.	16.24	857.6755 [M + HCOO]^−^	−0.04	C_47_H_93_N_2_O_6_P	281.2483, 797.6516	SM (d18:1/24:1)
191.	16.34	831.6603 [M + HCOO]^−^	0.73	C_45_H_91_N_2_O_6_P	281.2483, 783.6366	SM (d18:1/22:0)
192.	16.36	797.6538 [M − H]^−^	−1.71	C_46_H_91_N_2_O_6_P	797.6513 281.2481	SM (d18:2/23:0)
193.	16.43	769.6231 [M − H]^−^	0.35	C_44_H_87_N_2_O_6_P	305.2468, 283.2638	SM (d18:2/21:0)
194.	16.60	845.6759 [M + HCOO]^−^	−0.33	C_46_H_93_N_2_O_6_P	785.6514, 449.3144	SM (d18:1/23:0)
195.	16.70	859.6912 [M + HCOO]^−^	−0.14	C_47_H_95_N_2_O_6_P	799.6673	SM (d18:1/24:0)
196.	16.90	666.6046 [M + HCOO]^−^	0.53	C_40_H_79_NO_3_	338.3418, 321.3145, 263.2380, 237.2213, 620.5970, 364.3575	Cer (d16:1/24:0)
197.	16.95	692.6201 [M + HCOO]^−^	0.39	C_42_H_81_NO_3_	616.6036, 408.3846, 392.3885, 366.3727, 349.3473, 261.2226	Cer (d16:2/26:0)
198.	17.04	873.7067 [M + HCOO]^−^	−0.17	C_48_H_97_N_2_O_6_P	813.6832, 168.0417,78.9574,122.9837,449.3120	SM (d18:1/25:0)
199.	17.11	680.6200 [M + HCOO]^−^	0.12	C_41_H_81_NO_3_	634.6127, 604.6011, 378.3732, 352.3579, 355.3311, 263.2372	Cer (d16:1/25:0)
200.	17.37	694.6360 [M + HCOO]^−^	0.62	C_42_H_83_NO_3_	648.6278, 618.6174, 408.3834, 392.3886, 366.3724, 349.3455, 263.2383	Cer (d16:1/26:0)
201.	17.39	696.6513 [M + HCOO]^−^	0.58	C_42_H_85_NO_3_	649.6318, 619.6207, 409.3873, 393.3918, 367.3786, 350.3496, 263.2375	Cer (d16:0/26:0)
202.	17.45	708.6516 [M + HCOO]^−^	0.57	C_43_H_85_NO_3_	662.6441, 632.6330, 422.3997, 406.4042, 380.3884, 363.3622, 263.2374	Cer (d16:1/27:0)

### Pseudotargeted Lipidomics Analysis of Liver in IDILI Rats Caused by HSW

**FIGURE 6:**
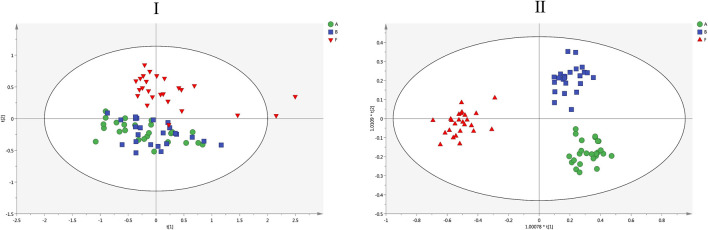
The score plots of PCA (I) and OPLS-DA (II) models for the 202 targeted lipidomics analysis. I: R2X and Q2 values of the PCA model are 0.798 and 0.636, respectively; II: R2Y and Q2 values of the OPLS-DA model are 0.913 and 0.772, respectively. Green circles, blue squares and red inverted triangles denote group control **(A)**, LPS **(B)** and LPS + H-HSW **(F)**, respectively.

A pseudotargeted lipidomics analysis of 202 identified features was further constructed to better understand the variation of the targeted lipid metabolism induced by HSW. The ion intensities of these lipids were extracted in a high-resolution, accurate-mass selected (HR/AM) mode. The peak areas of high-resolution selected ions vs. IS were used for relative quantitation, and 202 lipids were subjected to further multivariate statistical analysis. Firstly, the data matrix of 202 targeted lipids among the control, LPS and LPS + HSW groups were analyzed by using a PCA model. A PCA score plot (Figure 6I) showed that the liver samples from control and LPS groups were cluster together, while the LPS + HSW group showed a separation at a score of the t [2] component. Overall, the lipid metabolic changes observed in the lipidomics study were associated with the hepatotoxic result. The data matrix was then loaded into OPLS-DA model and profound disparities between the LPS + HSW group, and the other two groups were further displayed on the score plot of OPLS-DA (Figure 6II). It demonstrated that the alterations of metabolic pattern of given lipids was obviously induced by co-treated HSW with LPS.

**FIGURE 7:**
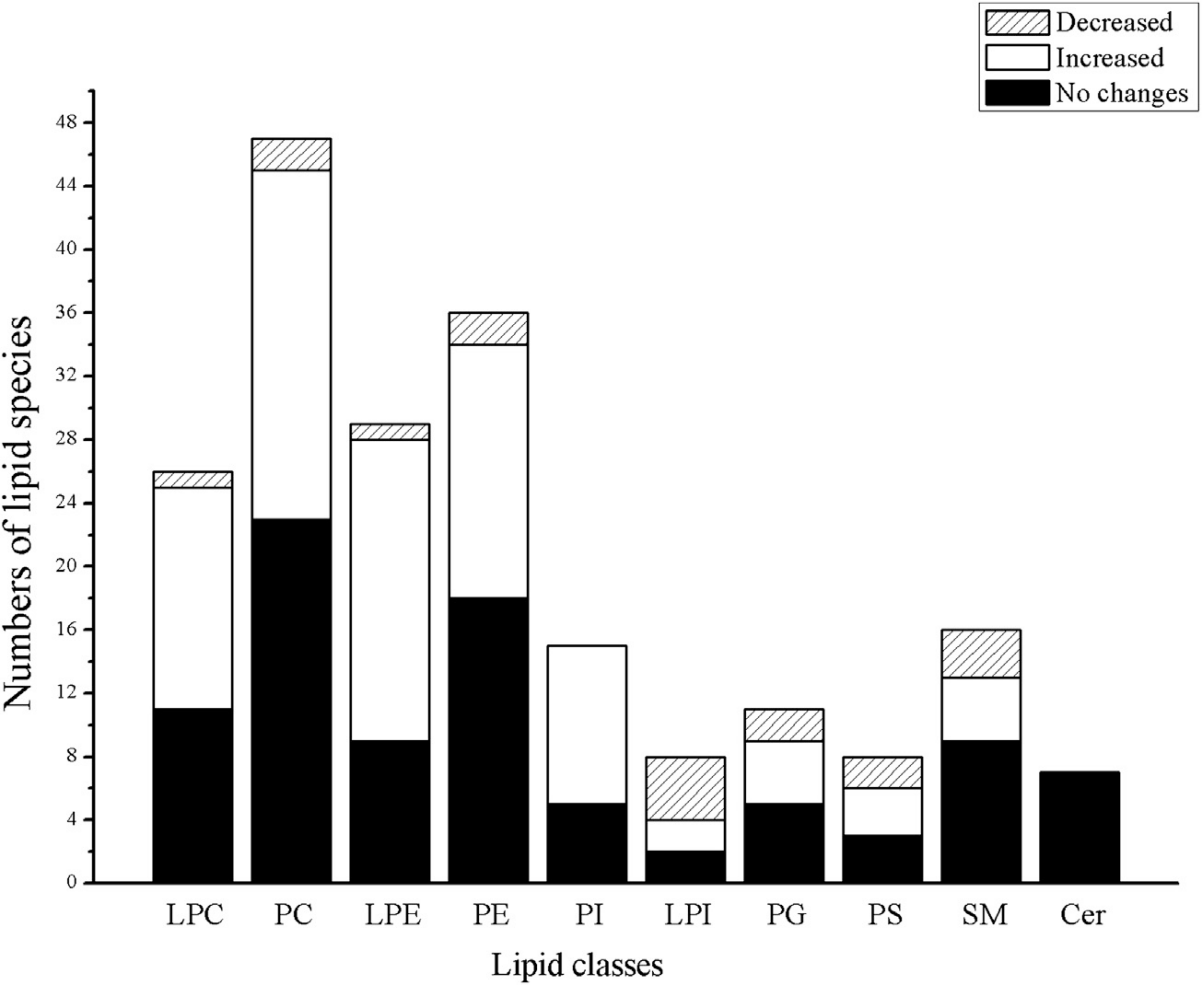
Histograms of the varied numbers of specific lipid classes between LPS + HSW and the LPS group.

A heat map showed 202 lipid variations of LPS and LPS + HSW compared to the control group (Supplementary Figure S4). Compared with the control group, the LPS model group did not showed distinct lipid variation, while 99 out of 202 lipids showed significant changes in LPS + H-HSW group (*p* < 0.05). To demonstrate the lipid alterations that respond for HSW-induced liver injury in the immune-stimulated idiosyncratic DILI rodent model, the comparison between the LPS + HSW and the LPS groups was further conducted. The variation of specific classes of lipid between the LPS + HSW and LPS group is summarized in Figure 7. Briefly, among the varied lipid species, 14 out 15 LPC, 22 out of 24 PC, 19 out of 20 LPE, 16 out of 18 PE, two out of three PS, and all the 10 PI were increasing with statistical significance with some exceptions.

Significant changed lipids in LPS + HSW group vs. the model group (fold change >1.5 and *p* < 0.001) are listed in Table 3, of which LPC, LPE, PC, and PE accounted for the majority. In animal tissues, PC and PE are the two most abundant glycerophospholipids (Drin, 2014). They are metabolized by phospholipases (PLA1 and PLA2) into arachidonic acids and LPC/LPE. The former is a key precursor of lipid pro-inflammatory and pro-resolving mediators that play pivotal roles in inflammation (Dennis and Norris, 2015). LPC, on the other hand, is an important mediator, the accumulation of which induces hepatocyte lipoapoptosis (Kakisaka et al., 2012), causes mitochondrial dysfunction (Hollie et al., 2014), and induces pro-fibrogenic extracellular vesicle (EV) release from hepatocytes (Ibrahim et al., 2016). An *in vitro* study proved that incubation of cultured hepatocytes with LPC triggered cell apoptosis (Donnelly et al., 2005). Besides, hepatic LPC content is increased in nonalcoholic steatohepatitis (NASH) and parallels liver disease severity (Puri et al., 2007; Zhou et al., 2016).

**TABLE 3:** Significant changed lipids for F group vs. the model group (fold change >1.5 and *p* < 0.001).

No	lipids class	name	*R*_t_ (min)	Fold-change	Response	*p* Value
1	LPC	LPC (20:5)	6.76	1.75	↑	0.0000
2	LPC	LPC (16:1)	7.17	1.52	↑	0.0000
3	LPC	LPC (18:2)	7.61	1.55	↑	0.0000
4	LPC	LPC (18:2)	7.84	1.54	↑	0.0000
5	LPC	LPC (18:1)	8.75	1.62	↑	0.0000
6	LPC	LPC (20:2)	8.77	1.77	↑	0.0000
7	LPC	LPC (20:1)	10.04	2.04	↑	0.0000
8	LPE	LPE (20:5)	7.08	1.76	↑	0.0000
9	LPE	LPE (20:5)	7.06	1.93	↑	0.0000
10	LPE	LPE (16:1)	7.44	1.89	↑	0.0000
11	LPE	LPE (20:2)	7.84	1.54	↑	0.0000
12	LPE	LPE (16:1)	7.16	1.77	↑	0.0000
13	LPE	LPE (18:2)	7.74	1.74	↑	0.0000
14	LPE	LPE (20:2)	7.55	1.57	↑	0.0000
15	LPE	LPE (18:2)	7.68	1.74	↑	0.0000
16	LPE	LPE (18:1)	8.48	1.73	↑	0.0000
17	LPE	LPE (18:1)	9.04	2.05	↑	0.0000
18	LPE	LPE (17:0)	9.26	1.71	↑	0.0000
19	LPE	LPE (20:1)	10.07	2.37	↑	0.0000
20	LPE	LPE (19:0)	10.33	1.74	↑	0.0000
21	PE	PE (18:3/18:2)	14.13	1.53	↑	0.0062
22	PE	PE (20:4/18:2)	14.36	1.57	↑	0.0045
23	PE	PE (20:5/16:0)	14.41	2.13	↑	0.0000
24	PE	PE (22:7/18:0)	15.02	0.65	↓	0.0000
25	PE	PE (18:1/16:0)	15.42	1.54	↑	0.0000
26	PE	PE (P-18:0/22:3)	16.10	2.03	↑	0.0000
27	PG	PG (22:6/16:0)	13.90	0.57	↓	0.0000
28	PG	PG (20:4/16:0)	14.04	0.66	↓	0.0033
29	PG	PG (18:2/18:2)	12.82	1.61	↑	0.0000
30	SM	SM (d18:1/15:0)	13.99	1.87	↑	0.0006
32	SM	SM (42:4)	15.36	0.34	↓	0.0000
33	LPI	LPI (18:4)	6.97	1.52	↑	0.0000
34	LPI	LPI (20:4)	7.05	0.72	↓	0.0001
35	PI	PI (20:4/18:1)	13.78	1.54	↑	0.0000
36	PI	PI (18:2/16:0)	13.68	1.53	↑	0.0021
37	PI	PI (20:3/16:0)	14.20	1.86	↑	0.0000
39	PI	PI (18:1/16:0)	14.22	1.54	↑	0.0007
40	PI	PI (18:2/18:0)	14.48	1.85	↑	0.0000
41	PC	PC (20:4/19:1)	15.01	1.51	↑	0.0000
42	PC	PC (20:3/18:0)	15.55	1.55	↑	0.0000
43	PC	PC (37:2)	15.68	1.54	↑	0.0000

As the main membrane phospholipid species, PC and PE serve pivotal biological functions involved in regulating lipoprotein metabolism (such as very-low-density lipoproteins in liver, VLDL) (Gibbons et al., 2004) and signaling via acting on G protein-coupled receptor and function in membrane fusion and fission (O’Donnell et al., 2019). The composition of PE and PC in cells varies considerably depending on the functional properties and physiological status of a tissue. Metabolism disorders of these lipids thus cause variation in the membrane lipid composition, which affects the membrane’s physical properties and functional integrity, resulting in hepatocyte apoptosis, inflammation, and liver disease progression (Li et al., 2006; Payne et al., 2014; Wu et al., 2019). Previous studies have reported variations in their contents, and the PE/PC ratio was associated with liver injuries induced by valproic acid (Goda et al., 2018), CCl_4_ (Shimizu, 1969; Sugano et al., 1970), tamoxifen (Saito et al., 2017) and APAP (Ming et al., 2017). Accumulation of these PC/PE could induce hepatocytes dysfunction. In the present study, PC, LPC, PE, LPE, and PI are the most increased lipid classes in the liver injury group, and they indicated that accumulation of these biological membrane lipids was associated with HSW-induced IDILI.

PI can be phosphorylated by kinases, such as PI-3-kinase (PI3K) and PI-4-kinase (PI4K), to produce a series of phosphoinositides (such as PI3P, PIP_2_, and PIP_3_), which function as signaling molecule in multiple pathways. PIP_2_ and PIP_3_ phosphorylate by PI3K can activate Akt, regulating cell survival, mitogenesis, and other cellular processes (Hemmings and Restuccia, 2015). Recent studies suggest the variations of PI in plasma and liver patients were associated with liver cirrhosis (Mcphail et al., 2016; Buechler and Aslanidis, 2020) and hepatocellular carcinoma (HCC) (Li et al., 2017b). In the present study, most of the detected PI in hepatocytes, including PI (18:2/18:0), PI (20:4/18:1), PI (20:3/16:0), and PI (18:2/16:0), were significantly increased in the liver injury group, and these could be used as potential biomarkers for diagnosis of HSW-induced hepatotoxicity. Whether or not the accumulation of these PI affects the liver cell growth and survival via the PI3K-AKT-mTOR pathway, though, deserves further study.

According to the clinical practice of traditional Chinese medicine, the dosage of HSW is equivalent to raw herb between 0.3 and 0.5 g/kg/day in most cases. The conventional experimental research on the toxic evaluation of HSW, however, requires as high a dosage as 50 g/kg/day (equivalent of raw herb) for 4–8 weeks (Fan et al., 2015). In the present study, two much lower dosages at 2 and 10 g/kg/day (equivalent of raw herb) were used in this MIS rat model; this is still out of range for a realistic dose (Heinrich et al., 2020). As a consequence, toxicity studies of HSW at a more therapeutically relevant dose are needed as a next step to explore the idiosyncratic property of HSW-induced liver injury in clinical practice.

## Conclusion

In this study, substantial liver damage caused by HSW in an LPS-induced rat model was confirmed by combination of an integrated morphological test, histological assessment, and biomedical analysis. A global analysis of 202 lipid metabolic variations in injured liver of rats induced by HSW was carried out based on an LC-MS lipidomics approach. Disturbed hepatic lipid homeostasis was observed, as PC, LPC, PE, LPE, and PI were increased in HSW-induced injured liver. Our results provide a better understanding of the role of disturbed lipid metabolism in HSW-induced injured liver, which might provide valuable information for clinical diagnosis of DILI and underlying mechanisms.

## Data Availability Statement

The raw data supporting the conclusions of this article will be made available by the authors, without undue reservation.

## Ethics Statement

The animal study was reviewed and approved by The laboratory animal ethics committee of Guangdong Province Hospital.

## Author Contributions

XW performed the investigation, formal analysis, data curation, and methodology; YZ performed the investigation, data curation, and validation; JQ, YX, and JZ worked on the investigation, visualization, and resources; ZH wrote, reviewed, and edited the manuscript; XQ worked on the conceptualization, investigation, resources; WX performed the conceptualization, wrote the original draft, performed project administration and funding acquisition and worked on the conceptualization and resources.

## Funding

The work was supported by Pearl River S&T Nova Program of Guangzhou (201806010048), National Natural Science Foundation of China (81803482), Science and Technology Planning Project of Guangdong Province, China (2016A020226037, 2016A020226045), and Special Subject of TCM Science and Technology Research of Guangdong Provincial Hospital of Chinese Medicine (YN2018QJ07, YN2016QJ01).

## Conflict of Interest

The authors declare that the research was conducted in the absence of any commercial or financial relationships that could be construed as a potential conflict of interest.
