# Supplementary material for: Lipidomics Analysis Indicates Disturbed Hepatocellular Lipid Metabolism in Reynoutria multiflora-Induced Idiosyncratic Liver Injury
Source: Front Pharmacol. 2020 Dec 21;11:569144. doi: 10.3389/fphar.2020.569144 (PMC7779765; doi:10.3389/fphar.2020.569144)
Supplement: Supplementary file 1 [file datasheet1.docx]

**Supplementary Information**

Chemical compositions of the HSW sample was analyzed by using a Ultra-high-pressure liquid chromatography coupled with a LTQ-Orbitrap mass spectrometer (UHPLC-LTQ-Orbitrap MS) method. The separation was performed by an Thermo Accela UHPLC instrument (Waltham, MA, USA) and the chromatography was carried out using a Thermo Hypersil GOLD C_18_ column (1.9 μm, 2.1 mm × 50 mm). The mobile phase was composed of acetonitrile (A) and water containing 0.1% formic acid (B) using the following gradient program: 5% A (0-1 min), 10% A (2 min), 20% A (3 min), 25% A (5 min), 50% A (20 min), 70% A (22-25), and 95% A (28-30). The flow rate was set at 200 μL/min. The column oven is set as room temperature. A LTQ-Orbitrap XL hybrid mass spectrometer (Thermo Fisher Scientific, Bremen, Germany) coupled with UHPLC instrument via an ESI interface. The conditions of ESI source were set as follows: sheath gas and auxiliary gas were 15 and 0 units, respectively; spray voltage was 3.8 kV; capillary temperature was 320 ºC; and tube lens voltage was 45 V. The Orbitrap mass analyzer was set up in negative ion mode and the full scan mass range was set from m/z 150 to 1200 at a resolution of 30 000 with the acquisition of centroided-type mass spectra. MS^2^ was acquired in data-dependent manner with the collision energy of 35% for CID. Dynamic exclusion parameters were set as follows: repeat count 2; repeat duration 7.5 s; exclusion duration 10 s. The identified components was listed in table S1 and the base ion chromatogram of HSW in negative mode was illustrated in Figure S1.

**Table S1** The main identified constituents in HSW by LC-MS.

| **No.** | ***R*t (min)** | **Precursor ions**  **[M-H]^-^** | **MS^n^ (negative mode)** | **Identification** | **types** |
| --- | --- | --- | --- | --- | --- |
|  | 1.65 | 191.01935  (C_6_H_7_O_7_) | 111(100), 173(15) | citric acid | organic acid |
|  | 7.63 | 577.13306  (C_30_H_25_O_12_) | 425(100), 407(53), 289(38), 451(23) | procyanidin B | tannins |
|  | 8.00 | 579.14893  (C_30_H_27_O_12_) | 289(100), 426(40), 407 (12), 469(9) | gambiriin A | tannins |
|  | 9.25 | 729.14307  (C_37_H_37_O_18_) | 577(100), 407(60), 559(38), 425(36), 289(16) | mono-*O*-galloylprocyanidin | tannins |
|  | 9.46 | 567.16992  (C_26_H_31_O_14_) | 405 (100) | 2,3,5,4′-tetrahydroxysilbence-2,3-glucoside | silbences |
|  | 11.12 | 405.11798  (C_20_H_21_O_9_) | 243(100) | 2,3,5,4′-tetrahydroxysilbence | silbences |
|  | 11.50 | 557.12854  (C_27_H_25_O_13_) | 313(100), 243(35), 405(19) | 2,3,5,4'-tetrahydroxysilbence-2-(galloyl)- glucoside | silbences |
|  | 12.48 | 447.12814  (C_22_H_23_O_10_) | 243(100) | 2,3,5,4'-tetrahydroxysilbence-2-(acetyl)-glucoside | silbences |
|  | 13.53 | 557.12830  (C_27_H_25_O_13_) | 313(100), 405(17), 243(14) | 2,3,5,4'-tetrahydroxysilbence-2-(galloyl)-glucoside | silbences |
|  | 14.49 | 447.09174  (C_21_H_19_O_11_) | 285(100) | citreorosein-*O*-glucoside | anthraquinones |
|  | 14.96 | 551.15399  (C_29_H_27_O_11_) | 405(100), 243(23), 307(15) | 2,3,5,4'-tetrahydroxysilbence-2-(coumaro-yl)-glucoside | silbences |
|  | 15.09 | 581.16479  (C_30_H_29_O_12_) | 405(100), 243(48), 387(32) | 2,3,5,4'-tetrahydroxysilbence-(feruloyl)- glucoside | silbences |
|  | 15.37 | 407.13336  (C_20_H_23_O_9_) | 245(100) | torachrysone-8-O-glucoside | naphthalenes |
|  | 15.76 | 431.09708  (C_21_H_19_O_10_) | 269(100) | emodin-8-*O*-glucoside | anthraquinones |
|  | 16.89 | 517.09723  (C_24_H_21_O_13_) | 473(100), 269(10) | emodin-8-*O*-(6′-*O*-malonyl)-glucoside | anthraquinones |
|  | 24.82 | 269.04474  (C_15_H_9_O_5_) | 225(100), 241(23) | emodin | anthraquinones |

**Figure S1**. The base ion chromatogram (BIC) of the root of *Reynoutria multiflora* (HSW) in negative mode of LC-Orbitrap MS.


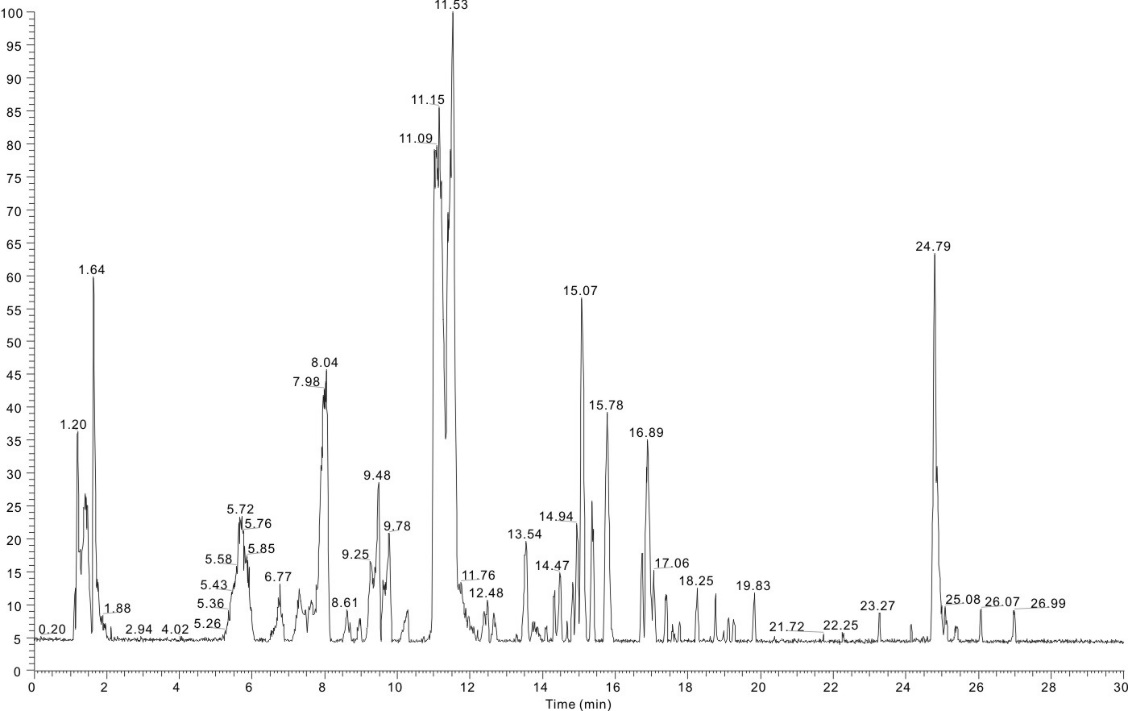


Figure S2. The total ion chromatograms (TIC) of the control, LPS and LPS+H-HSW samples in negative and positive modes.


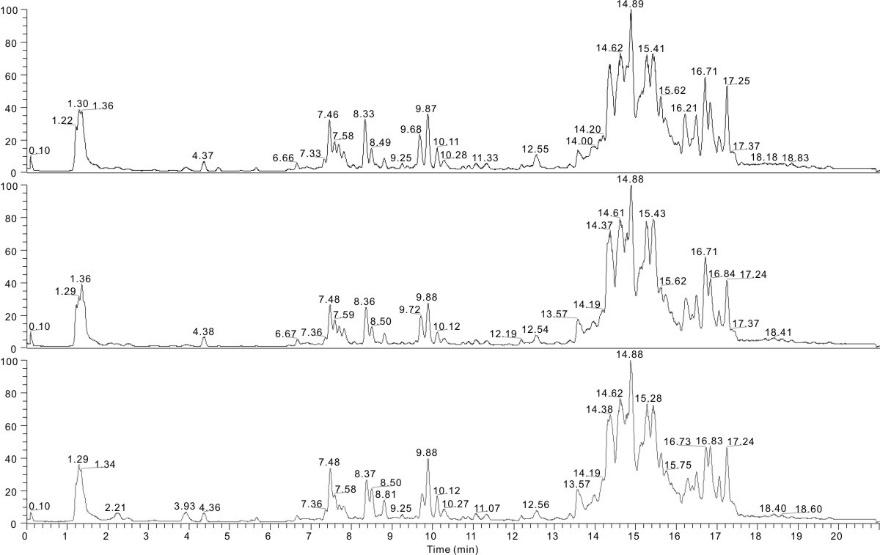


**Negative mode**

**control**

**LPS**

**LPS+H-HSW**


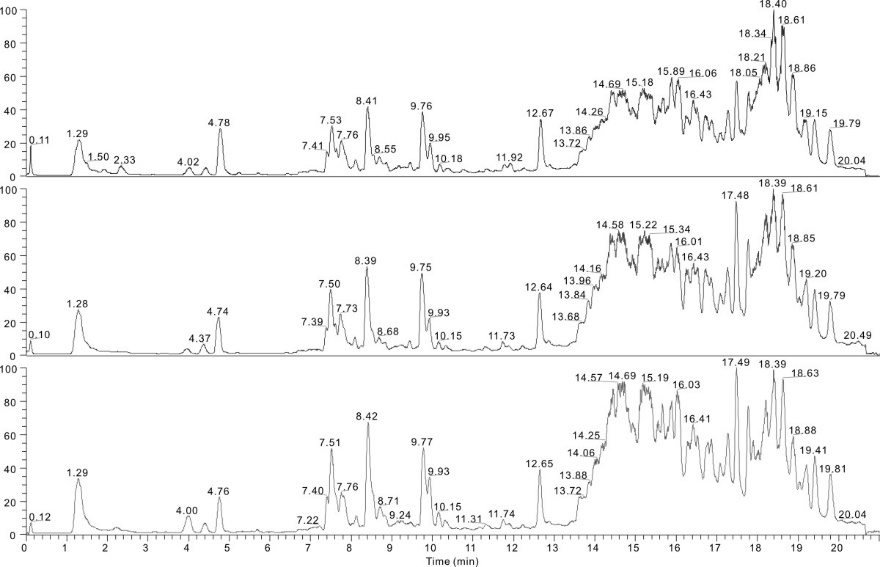


**Positive mode**

**control**

**LPS**

**LPS+H-HSW**

**Figure S3**. The Permutations tests of 200 times for the OPLS-DA model of the untargeted lipidomic analysis (A) and Pseudotargeted lipidomic analysis (B).


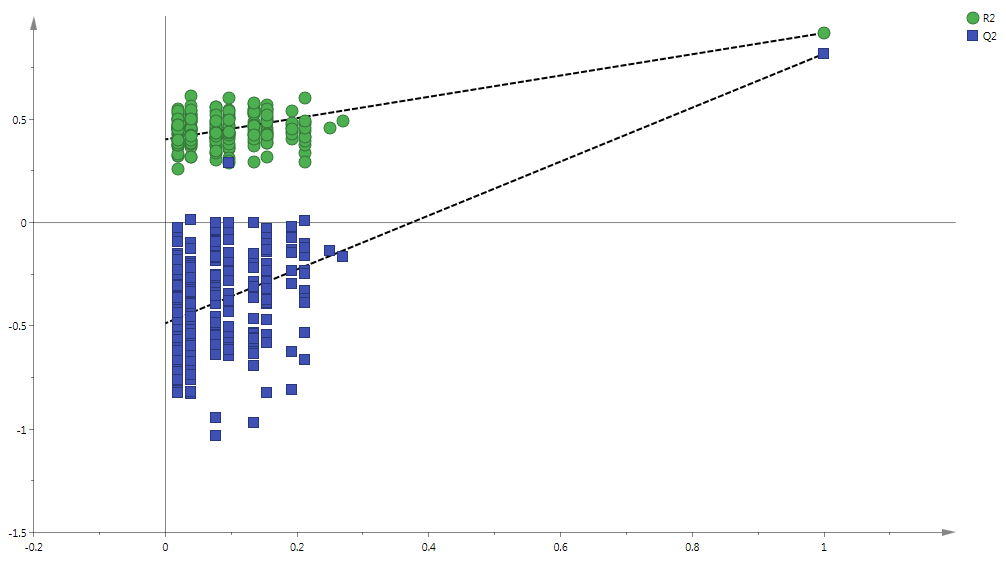


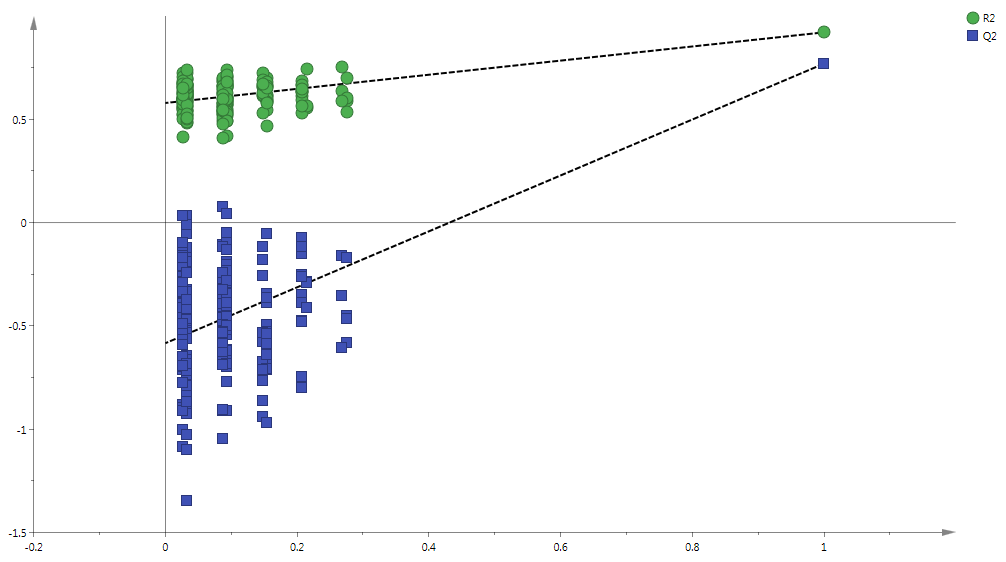


A B


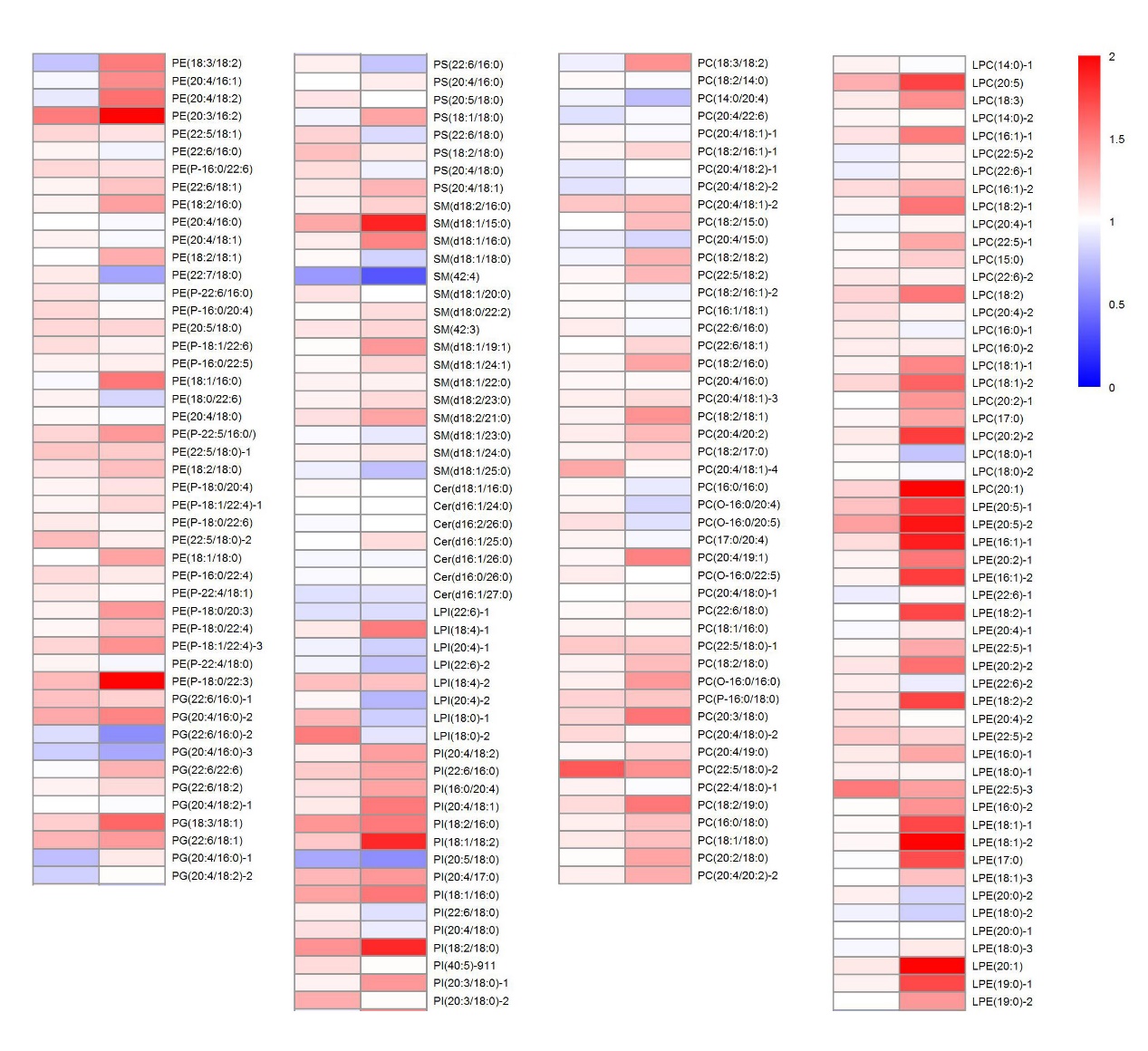


**Figure S4.** Heat map of the 202 lipid variations of LPS and LPS+HSW against control group. (Left: LPS against control; right: LPS+H-HSW against control. Fold changes < 1, =1 and > 1 were expressed as blue, white and red, respectively.)
